# The *nphp-2* and *arl-13* Genetic Modules Interact to Regulate Ciliogenesis and Ciliary Microtubule Patterning in *C. elegans*

**DOI:** 10.1371/journal.pgen.1004866

**Published:** 2014-12-11

**Authors:** Simon R. F. Warburton-Pitt, Malan Silva, Ken C. Q. Nguyen, David H. Hall, Maureen M. Barr

**Affiliations:** 1 Department of Genetics, Rutgers, The State University of New Jersey, Piscataway, New Jersey, United States of America; 2 Center for *C. elegans* Anatomy, Albert Einstein College of Medicine, Bronx, New York, New York, United States of America; Washington University School of Medicine, United States of America

## Abstract

Cilia are microtubule-based cellular organelles that mediate signal transduction. Cilia are organized into several structurally and functionally distinct compartments: the basal body, the transition zone (TZ), and the cilia shaft. In vertebrates, the cystoprotein Inversin localizes to a portion of the cilia shaft adjacent to the TZ, a region termed the “Inversin compartment” (InvC). The mechanisms that establish and maintain the InvC are unknown. In the roundworm *C. elegans,* the cilia shafts of amphid channel and phasmid sensory cilia are subdivided into two regions defined by different microtubule ultrastructure: a proximal doublet-based region adjacent to the TZ, and a distal singlet-based region. It has been suggested that *C. elegans* cilia also possess an InvC, similarly to mammalian primary cilia. Here we explored the biogenesis, structure, and composition of the *C. elegans* ciliary doublet region and InvC. We show that the InvC is conserved and distinct from the doublet region. *nphp-2* (the *C. elegans* Inversin homolog) and the doublet region genes *arl-13*, *klp-11,* and *unc-119* are redundantly required for ciliogenesis. InvC and doublet region genes can be sorted into two modules—*nphp-2*+*klp-11* and *arl-13*+*unc-119—*which are both antagonized by the *hdac-6* deacetylase. The genes of this network modulate the sizes of the NPHP-2 InvC and ARL-13 doublet region. Glutamylation, a tubulin post-translational modification, is not required for ciliary targeting of InvC and doublet region components; rather, glutamylation is modulated by *nphp-2*, *arl-13*, and *unc-119*. The ciliary targeting and restricted localization of NPHP-2, ARL-13, and UNC-119 does not require TZ-, doublet region, and InvC-associated genes. NPHP-2 does require its calcium binding EF hand domain for targeting to the InvC. We conclude that the *C. elegans* InvC is distinct from the doublet region, and that components in these two regions interact to regulate ciliogenesis via cilia placement, ciliary microtubule ultrastructure, and protein localization.

## Author Summary

Cilia are sensory organelles that are found on most types of human cells and play essential roles in diverse processes ranging from vision and olfaction to embryonic symmetry breaking and kidney development. Individual cilia are divided into multiple functionally and compositionally distinct compartments, including a proximal “Inversin” compartment, which is located near the base of cilia. We used the nematode *C. elegans*, a well-defined animal model of cilia biology, to characterize the genetics, components, and defining properties of the proximal cilium. The Inversin compartment is conserved in *C. elegans*, and is established independent of another proximal ciliary region, the microtubule doublet-based region. We showed how components of both the doublet region and the Inversin compartment genetically interact to regulate many pathways linked to core aspects of cilia biology, including ciliogenesis, cilia placement, cilia ultrastructure, microtubule stability, and the protein composition of ciliary compartments. In addition to expanding and clarifying our knowledge of basic cilia biology, these results also have direct implications for human health research because several of the genes and pathways explored in our work are linked to ciliopathies, a group of diseases caused by dysfunctional cilia.

## Introduction

Cilia are cellular “antennae” that mediate the transduction of environmental signals into intracellular pathways. Cilia play an integral role in many cellular functions, including developmental signaling, symmetry breaking, cell-cell adhesion, cell-cycle control, stress response, and DNA damage response (e.g., [1]–[6]). The vast majority of cilia share a set of evolutionarily conserved features: cilia are supported by a microtubule-based backbone, the axoneme; are built by intraflagellar transport (IFT), a microtubule motor driven cargo transport system [7]; and can be divided into structurally and functionally distinct compartments. These compartments include the microtubule triplet basal body which roots the cilium to the cell, the microtubule doublet transition zone (TZ) which anchors the cilium to the membrane, and the microtubule doublet cilia shaft where IFT occurs. The basal body and TZ also act as selective filters for inbound and outbound ciliary cargo, functioning through physical occlusion and cargo-specific recognition mechanisms [7]–[9]. The cilia shaft has traditionally been treated as an undifferentiated whole [10], though recent evidence has shed light on subdivisions of the cilia shaft [11].

Inversin/Nephrocystin-2 specifically localizes to the Inversin compartment (InvC), a proximal portion of the cilia shaft adjacent to the TZ [12]. This region has been suggested to play a role in signal transduction and amplification [13]–[15], cilia placement, and ciliogenesis [16], [17]. Products of several genes—including *INVS/NPHP2*, *NPHP3*, *NEK8/NPHP9*, and *ANKS6/NPHP16*—localize to the InvC. Interactions between InvC genes and other cilia genes have only recently begun to be explored and have not been well generalized across animal and cell culture models [18]–[22].

The mechanisms that initially establish the InvC are currently unknown, though recruitment pathways for several InvC components are known. Work in vertebrate models has shed light on an InvC-specific physical interaction complex composed of Inversin, Nek8, Nphp3, and Anks6 [23]–[25]. Nek8, Nphp3, and Anks6 localize to the InvC in an Inversin-dependent manner, but Inversin itself localizes independently of the other proteins [23], [24]. Unc119b—a possible, though not proven, InvC component—may mediate an InvC-targeting pathway. In mammalian cells, Unc119b binds myristoylated cargo, including Nphp3, and shuttles it into the cilium. Once the Unc119b-Nphp3 complex has translocated into the cilium, the small GTPase Arl3 triggers Unc119b to release bound cargo [26]. InvC components other than Nphp3 are not known to be myristoylated and shuttled via Unc119b, suggesting additional InvC targeting pathways must exist. Whether Unc119b is required for the localization of Inversin and how Unc119b itself is targeted to the proximal cilium is unknown.

The nematode *Caenorhabditis elegans* is a well-studied model of cilia biology [27]. *C. elegans* possesses a ciliated nervous system [28]–[31] which is primarily used by the roundworm to detect internal and external cues and signals. Amphid channel cilia in the head and phasmid cilia in the tail are exposed to and sense the external environment through cuticular pores [32], [33]. Unlike most mammalian primary cilia, the cilia shafts of *C. elegans* amphid channel and phasmid cilia are divided into two regions: a proximal microtubule doublet-based region attached to the TZ, and a distal microtubule singlet-based region that extends from the doublet region [32], [33]. As both the doublet region of *C. elegans* cilia and the InvC of mammalian primary cilia lie at the proximal end of the cilium, directly adjacent to the TZ at the cilia base, and constitute only a portion of the length of the cilia shaft, previous work has viewed them as compositionally and functionally similar [13], [17], [34]. The relationship between the mammalian InvC and the *C. elegans* doublet region of cilia has not been well characterized; Here we present evidence that the InvC and the doublet region are distinct, but overlapping, ciliary regions.

The *C. elegans* genome encodes orthologs for several of the mammalian InvC-associated proteins, including Inversin itself (NPHP-2), Unc119b (UNC-119), Arl3 (ARL-3), and possibly Nek8 (the uncharacterized paralogous pair NEKL-1 and NEKL-2), but likely not Nphp3 or Anks6. Of these, NPHP-2 and ARL-3 have previously been shown to be doublet region-localizing in *C. elegans* [17], [35]. *C. elegans* also possess several doublet region-enriched proteins, which are not InvC restricted in mammalian primary cilia; these include the kinesin-II IFT motor KLP-11 and the membrane-associated small GTPase ARL-13 [35]. The IFT motors Kinesin-II and OSM-3 work cooperatively to carry the IFT assemblies IFT-A and IFT-B and to build the doublet region—OSM-3 alone is sufficient to build the singlet region [36]. ARL-13 likely stabilizes the interaction between IFT-A and IFT-B particles and is required for ultrastructural integrity of the doublet region [35], [37]. *arl-13* mutants exhibit multiple ciliary defects, some of which can be suppressed by deletion of histone deacetylase *hdac-6*, through an unknown mechanism [35]. Mammalian *HDAC6* also antagonizes ciliogenesis in mammalian primary cilia: *HDAC6* knockouts can suppress ciliogenesis defects arising from *INVS/NPHP2* RNAi in MDCK cells [16].

In this work, we aimed to molecularly dissect the proximal cilium of *C. elegans* and to gain insight into the nature of the InvC and doublet region. We examined interactions between genes associated with the doublet region, determined the territories and localization dependencies of the protein products of these genes, and performed ultrastructural analysis of deletion mutants of these genes. We find that the InvC is conserved in *C. elegans*, is established early in development, and is distinct from the doublet region. *nphp-2* interacts with doublet region genes to regulate cilia placement, microtubule ultrastructural patterning, tubulin glutamylation, and territory sizes of NPHP-2 and ARL-13. Finally, we show that *nphp-2*, *arl-13*, *klp-11*, and *unc-119* fall into two parallel redundant genetic modules, and that interactions between the two modules are modulated by *hdac-6* and *arl-3*. Together, the InvC and the doublet region function in concert to regulate many critical aspects of ciliogenesis and cilia biology.

## Results

### Genetic interactions between *nphp-2* and *arl-13* are modulated specifically by *hdac-6*

**Figure 1:**
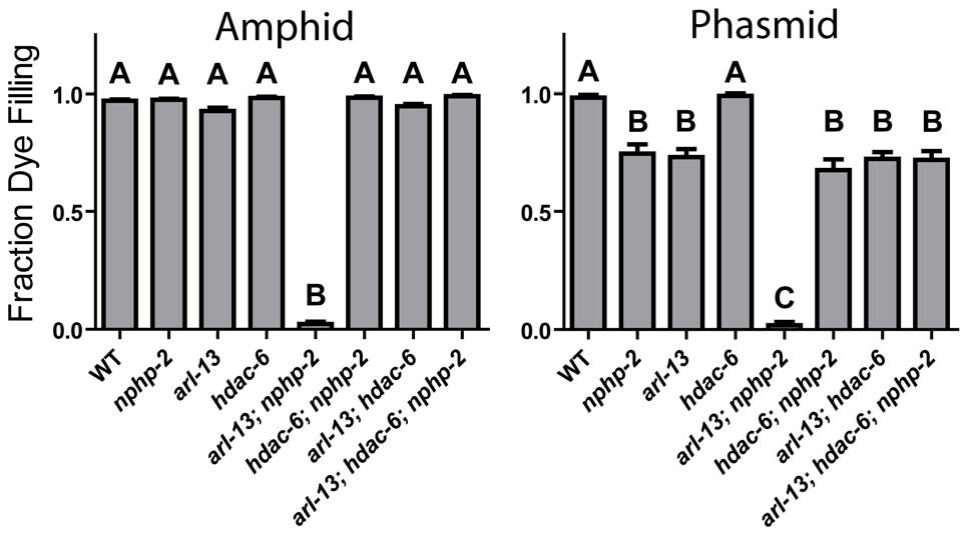
The synthetic dye-filling defective phenotype of *arl-13; nphp-2* mutants is modulated by *hdac-6*. *hdac-6* deletion suppresses *arl-13; nphp-2* double mutant phenotypes. In phasmids, *nphp-2* and *arl-13* single mutants are mildly Dyf, which is not suppressed by *hdac-6* deletion. In both amphids and phasmids, *arl-13; nphp-2* is severely SynDyf and was suppressed by *hdac-6*. Data in both panels was analyzed with pairwise Mann-Whitney U-test between all groups, followed by the Holm-Bonferroni multiple comparison adjustment with a total alpha of 0.01. Groups from either panel sharing a capital letter are not significantly different, whereas groups from either panel with different capital letters do differ significantly.

*nphp-2* and *arl-13* single mutants have statistically similar, moderate ciliogenic defects (Fig. 1). As *hdac-6* and *arl-3* modulate several *arl-13* phenotypes [35], we sought to determine if *nphp-2* and *arl-13* genetically interact and if *hdac-6* and *arl-3* modulate *nphp-2* phenotypes. We examined double, triple, and quadruple mutant combinations using “dye filling” of ciliated neurons as a gross indicator of ciliogenesis and cilia integrity [32]. Properly formed and placed cilia are environmentally exposed and take up fluorescent DiI dye, whereas stunted and misplaced cilia are not exposed and cannot take up DiI. Unlike the mild dye-filling defects (Dyf) of *nphp-2* and *arl-13* single mutants, *arl-13; nphp-2* double mutants were severely synthetic dye-filling defective (SynDyf) in both the amphids and phasmids (Fig. 1). *hdac-6* deletion did not suppress *nphp-2* or *arl-13* Dyf; this is contrary to previously published data indicating that *hdac-6* can partially suppress the weak *arl-13* single mutant Dyf, and may be due to a difference in dye filling or scoring method [35] (Materials and Methods). However, *hdac-6* suppressed the *arl-13; nphp-2* severe SynDyf phenotype to the mild Dyf severity of the single mutants in both amphids and phasmids (Fig. 1). *hdac-6* may function by suppressing defects arising from one of the pathways or at a point where the two pathways converge. Combined, this data indicates that *arl-13* and *nphp-2* act in partially redundant parallel pathways antagonized by *hdac-6*.

*arl-3* has also been implicated as a modulator of the *arl-13* pathway [35]. We found that, unlike the interactions with *hdac-6*, interactions with *arl-3* are cell-type specific (S1A Figure). Both *arl-3* single mutants and *arl-3; hdac-6* double mutants were nonDyf in both amphids and phasmids. In amphids, *arl-13; arl-3* was mildly SynDyf, whereas *arl-3; nphp-2* is nonDyf (cf. Fig. 1 and S1A Figure). *hdac-6* deletion suppressed the *arl-13; arl-3* phenotype to a severity similar to that of the *arl-13* single mutants. In phasmids, both *arl-13; arl-3* and *arl-3; nphp-2* double mutants exhibited a moderate SynDyf phenotype. *hdac-6* suppressed *arl-13; arl-3* defects, but not *arl-3; nphp-2* defects. Strikingly, in both amphids and phasmids, *arl-3* deletion prevented the *hdac-6* Dyf suppression in the *arl-13; hdac-6; nphp-2* triple mutant described above. The SynDyf phenotype of *arl-13; arl-3* is qualitatively different from the suppression of *arl-13* Dyf defects by *arl-3* found previously [35]. Similarly to the difference in *hdac-6* suppression of *arl-13* defects discussed above, this may be due to different scoring methods or assay conditions. We conclude that *arl-3* functions parallel to both *nphp-2* and *arl-13* pathways, and likely lies in the same regulatory pathway as, but acts antagonistically to, *hdac-6*.

### *nphp-2* and *arl-13* genetically interact to regulate amphid cilia ultrastructure

**Figure 2:**
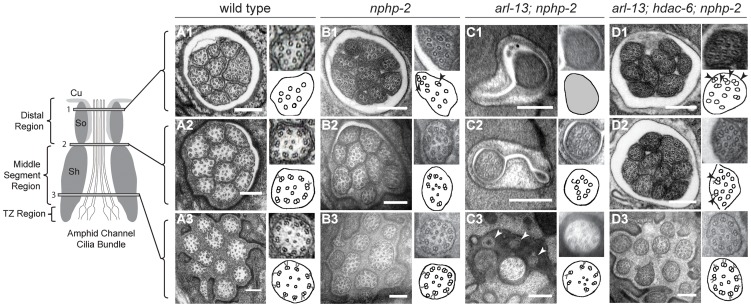
*nphp-2* single, *arl-13; nphp-2* double, and *arl-13; hdac-6; nphp-2* triple mutants exhibit defects in ciliary ultrastructure. Horizontal panels indicated by 1, 2, and 3 correspond to singlet region, doublet region-to-singlet region transition, and TZ levels, respectively, and are comparable across the genotypes. The insets show an enlarged view of the region within the white box, and are diagrammed in the accompanying cartoon. All scale bars are 250 nm. (A1–3) In wild-type amphids, all doublet B-tubules within a cilium have similar spans. (A1) Distal microtubule singlets are devoid of B-tubules. B-tubule containing microtubule doublets are present in (A2) the doublet region and (A3) the TZ. (B1–3) In *nphp-2* animals, amphid channel cilia are shifted lengthwise with respect to each other (cf. Fig. 6A). Within a cilium, spans of microtubule doublet B-tubules are asynchronous; arrows in insets indicate these microtubules. TZ Y-links are disorganized (C1–3) In *arl-13; nphp-2* animals, most amphid channel cilia are absent. (C1) The distal end is filled with electron dense material with unresolvable microtubules. (C2) A single, stub-like cilium is visible, consisting of only microtubule singlets. (C3) TZ microtubules are abnormal with some missing microtubule doublets. We also observe vesicle-like structures at this level that are indicated by arrows. (D1–3) In *arl-13; hdac-6; nphp-2* animals, most amphid cilia are visible. Ectopic singlets and doublets are still present. Cilia are shifted posteriorly towards the tail. (D1–2) insets show asynchronous microtubules within cilia.

To gain a better understanding of the defects present in *nphp-2* single and *arl-13; nphp-2* double mutants, and to examine the effects of *hdac-6* mediated suppression of the double mutant defects, we used serial-section transmission electron microscopy (TEM) to examine the ultrastructure of amphid channel cilia. Wild-type amphid cilia are divided into three segments based on microtubule ultrastructure: the TZ, the doublet region, and the singlet region (Fig. 2A). One of the two microtubules within a doublet, the A-tubule, extends to form the microtubule singlet seen in the distal cilium (Fig. 2A1); the second tubule of the doublet, the B-tubule, terminates at the distal end of the doublet region. The doublet microtubules of the TZ and doublet region are arranged in a circular pattern in close proximity to the ciliary membrane (Fig. 2A2, 2A3). Within a particular cilium, microtubule ultrastructural characteristics—the location in the lumen, membrane association, and singlet/doublet architecture—are similar across all nine outer microtubule doublets.

In *nphp-2* single mutants, in a given amphid cross-section at a single level, we observe singlet regions of some cilia, doublet regions of other cilia, and TZs of the remaining cilia. This is consistent with amphid cilia that were shifted lengthwise with respect to each other, indicating a potential anchoring defect (Fig. 2B3). Additionally, within a given cilium, doublet and singlet microtubule spans were no longer aligned. In sections across the proximal axoneme, this appeared as singlets amongst the expected doublets, and in sections across the distal axoneme, this appeared as doublets interspersed between the expected singlets (Fig. 2B1–3). These defects indicate that *nphp-2* is required both for microtubule patterning and for cilia anchoring. While the chemical fixation method utilized here does not preserve Y-link ultrastructure well, *nphp-2* mutants exhibited significantly greater Y-link disorder than observed in wild-type animals. The TZ defects seen in *nphp-2* animals may be related to both the lengthwise cilia shift and the TZ-placement defect previously reported in *nphp-2* mutants [17]. This set of defects has not been reported in any other *C. elegans* cilia mutant, suggesting that *nphp-2* functions in a novel capacity.

*arl-13* single mutants also exhibit a range of ultrastructural defects [35], [37]. Doublets are observed in the central lumen of the cilium, which may originate from either displaced outer doublets or mispatterned inner singlets. In both *C. elegans arl-13* and *hennin/ARL13B* mouse mutants, there is also an increased frequency of early B-tubule detachment from the A-tubule, indicative of microtubule stability or patterning defects [38]. Similar to *nphp-2* mutants, ectopic microtubule singlets were sometimes visible in the doublet region of *arl-13* worms [35], [37]. *arl-13; nphp-2* double mutants exhibited extreme ultrastructural defects, likely causative of the severe Dyf phenotype (Fig. 2C1–3). Cilia were almost completely absent from the amphid channel pore. No other cilia were visible even at the distal dendritic level. In one instance, a single cilium was visible in TEM sections. At the TZ level, an incomplete set of doublets was visible within the single cilium (Fig. 2C3). Closer to the socket cell/sheath cell transition, a set of singlets was visible (Fig. 2C2). At the distal pore, we were unable to resolve any internal structure due to electron dense material filling the cilium (Fig. 2C1).

Remarkably, in *arl-13; hdac-6; nphp-2* triple mutants almost all defects observed in the double mutant were suppressed (Fig. 2D1–3). The only defects remaining were misplaced TZs (Fig. 2D3), ectopic singlets in the doublet region (Fig. 2D2), and ectopic doublets in the singlet region (Fig. 2D1), similar to those present in the *nphp-2* single mutant. We did not observe inner doublets as reported in *arl-13* single mutants [35], [37]. These results are consistent with the observed *hdac-6* suppression of *arl-13; nphp-2* Dyf defects.

Because *nphp-2* and *arl-13* both exhibit ectopic microtubule singlets, *nphp-2* mutants exhibit ectopic microtubule doublets, and *nphp-2; arl-13* double mutants exhibit severe defects in ciliogenesis, we conclude that *nphp-2* and *arl-13* function together redundantly in regulation of microtubule patterning and ciliogenesis. Because ciliogenic defects are suppressed by *hdac-6*, but ectopic doublets and singlets are not, ciliogenesis and microtubule patterning may be independently regulated.

### NPHP-2 and ARL-13 do not require TZ- and doublet region-associated genes for ciliary targeting

**Figure 3:**
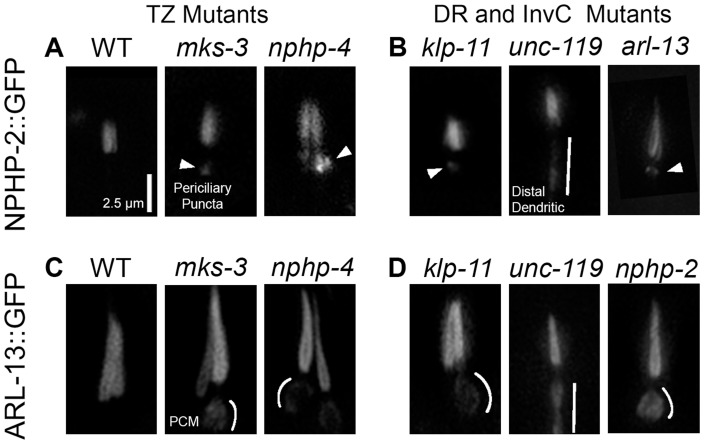
NPHP-2 and ARL-13 do not require TZ-, doublet region-, and InvC-associated genes for ciliary targeting. (A) In wild type (WT), NPHP-2 is restricted to the proximal cilium. In both *nphp-4* and *mks-3* mutants NPHP-2::GFP was targeted to the cilium and restricted to the post-TZ proximal cilium. Several NPHP-2::GFP puncta were visible in the periciliary compartment. (B) In doublet region and InvC mutants, NPHP-2::GFP was targeted to the cilium and restricted to the post-TZ proximal cilium in *klp-11*, *arl-13*, and *unc-119* mutants. *arl-13* and *klp-11* mutants exhibited periciliary NPHP-2::GFP puncta, and *unc-119* mutants exhibited distal dendritic accumulation of NPHP-2::GFP. (C) In WT, ARL-13::GFP localizes to the proximal cilium. In both *nphp-4* and *mks-3* mutants, ARL-13::GFP was targeted to the cilium and restricted to the post-TZ proximal cilium. ARL-13::GFP also mislocalized to the periciliary membrane compartment in TZ mutants. (D) In *nphp-2* and *klp-11* mutants, ARL-13::GFP was targeted to the cilium and restricted to the post-TZ proximal cilium. In these mutants, ARL-13::GFP also mislocalized to the periciliary membrane compartment, and in *unc-119* mutants, ARL-13::GFP mislocalized to the distal dendrite. Periciliary puncta – arrowheads, periciliary membrane – white arc, distal dendrite/periciliary accumulation – white bar. Periciliary membrane localization was judged by a visible enrichment of ARL-13::GFP on the edges of the periciliary compartment without a concomitant enrichment in the interior lumen of the periciliary region.

Localization of InvC and doublet region components can be broken down into three steps: first the protein is targeted to the cilia base, second, the protein is imported into the cilium, and third, the protein is restricted to a subdomain of the cilium [23]. The factors required for the initial establishment of the InvC and doublet region cilia targeting and localization restriction are unknown. The TZ functions as a regulator of ciliary protein import (Reviewed in [39]), and has been implicated in the import of InvC and doublet region components in mammalian cilia [40], [41]. As both *nphp-2* and *arl-13* genetically interact with TZ-associated genes [17], [37], we wanted to determine if NPHP-2 and ARL-13 ciliary targeting and import requires TZ components. In *C. elegans*, TZ genes are organized into two genetic and physical modules—the *mks* module and the *nphp-1*+*nphp-4* module [8], [17], [25], [42]. We examined the localization of NPHP-2 and ARL-13 in mutants missing a component of each module (Fig. 3A,C).

**Figure 4:**
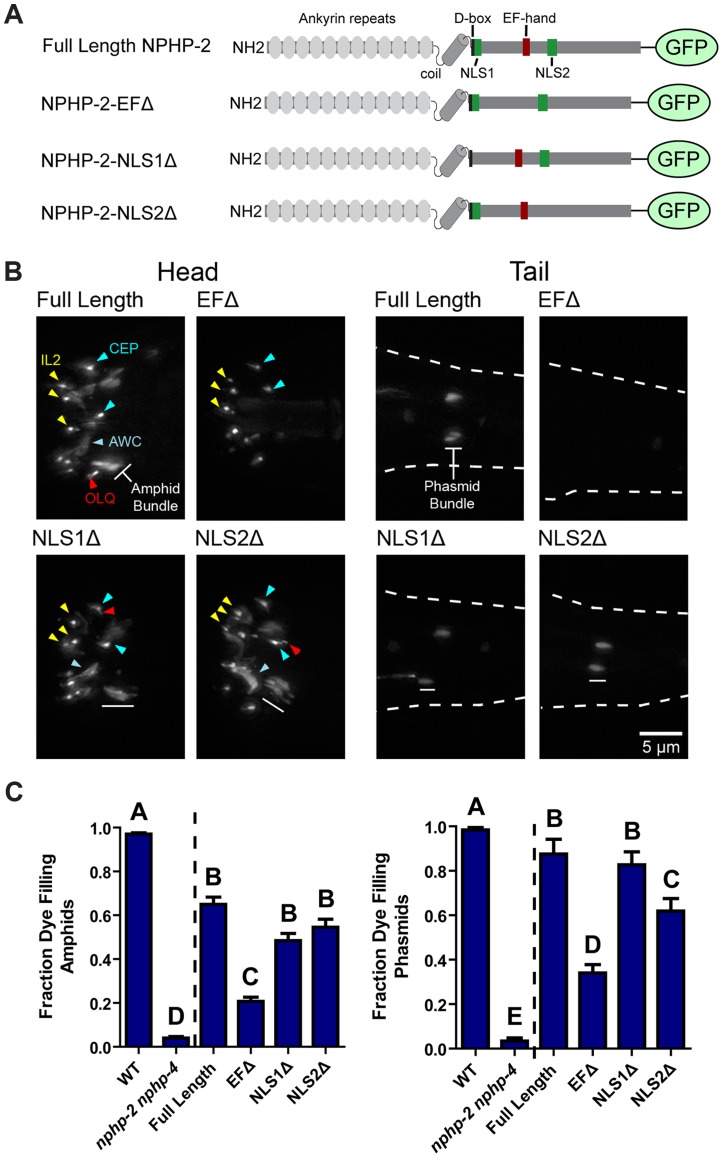
The EF hand is necessary for proper NPHP-2 localization and function. (A) Diagram of the domains present in Inversin, NPHP-2, and each of the NPHP-2 domain deletion constructs. (B) Localization of GFP-tagged NPHP-2 domain deletion constructs. In the head, full length NPHP-2::GFP localizes to the InvC of amphid channel cilia, to the base of IL2 cilia, and to the base of either OLQ or CEP cilia. In the tail (outlined by dashes), NPHP-2::GFP localizes to the InvC of phasmid cilia. Localization of both NPHP-2-NLS1Δ::GFP and NPHP-2-NLS2Δ::GFP appeared roughly wild-type in all cell types, though both constructs appeared enriched in AWC wing cilia. NPHP-2-EFΔ::GFP is present in IL2 and CEP cilia but fails to localize to amphid channel and phasmid cilia. (C) NPHP-2-NLS1Δ::GFP, NPHP-2-NLS2Δ::GFP, and NPHP-2-EFΔ::GFP can rescue *nphp-2 nphp-4* amphid and phasmid dye-filling defects, but NPHP-2-EFΔ::GFP rescue is significantly worse than full length NPHP-2::GFP in both amphid and phasmid neurons. Yellow arrowheads - IL2 cilia, red arrowheads - OLQ, blue arrowheads - CEP cilia, white bar - amphid/phasmid bundle. Letters indicate statistically distinct groups. Data was analyzed with pairwise Mann-Whitney U-test against both positive and negative controls, followed by the Holm-Bonferroni multiple comparison adjustment with a total alpha of 0.01. Non-transgene expressing siblings were used as negative controls in rescue experiments.

NPHP-2::GFP signal length is similar in wild-type and *nphp-2* backgrounds (1.77±0.23 um vs 1.74±0.22 um, st. dev.) and NPHP-2::GFP rescues the SynDyf phenotype of the *nphp-2 nphp-4* double mutant (Fig. 4B) [17]. These results indicate that this reporter reflects NPHP-2 functional and endogenous localization. In both *nphp-4* and *mks-3* single mutants, NPHP-2::GFP was properly targeted to and imported into the cilium. Mislocalized NPHP-2::GFP puncta in the periciliary region were sometimes visible (Fig. 3A). In *mks-3; nphp-4* double mutants, there were severe ciliogenic and dendritic extension errors, as previously reported [17], [42]; in phasmid cilia that were visible and placed properly, NPHP-2::GFP localization appeared as in *mks-3* and *nphp-4* single mutants (S2A Figure). In a wild-type background, ARL-13::GFP localized exclusively to the doublet region in amphid channel and phasmid neurons (Fig. 3C). Like NPHP-2::GFP, ARL-13::GFP was targeted to and imported into the cilium properly in *mks-3* and *nphp-4* mutants. Similar to published reports, we also observed ARL-13::GFP mislocalization to the periciliary membrane, as judged by a fluorescent “fringe” surrounding the periciliary region where the membrane lies (Fig. 3C, enhanced contrast in S2B Figure), which has been suggested to be due to a failure of the TZ diffusion barrier [34]. In amphid cilia, like in phasmid cilia, both NPHP-2 and ARL-13 were targeted to the cilium and imported properly, and both infrequently exhibited mild mislocalization (S3 Figure).

We investigated whether doublet region-associated genes were required for doublet region restriction of NPHP-2::GFP and ARL-13::GFP. *hdac-6* had no obvious effect on the localization of NPHP-2::GFP (S2A Figure). NPHP-2::GFP was targeted and restricted to the proximal cilium in both *klp-11* and *arl-13* mutants. In both mutants, periciliary puncta similar to those seen in TZ-associated mutants were visible (Fig. 3B). *unc-119* mutants also exhibited proper NPHP-2::GFP ciliary targeting. In *unc-119* mutants, NPHP-2::GFP exhibited a unique distal dendrite mislocalization pattern, distinct from the periciliary puncta seen in other TZ and doublet region mutants (Fig. 3B).

In *klp-11* and *nphp-2* mutants, ARL-13::GFP was restricted to a proximal portion of the cilium as in wild type, but with a periciliary membrane mislocalization pattern similar to TZ mutants. ARL-13::GFP also did not require *unc-119* for ciliary localization. In *unc-119* mutants, ARL-13::GFP and NPHP-2::GFP localized along the distal dendrite in a similar manner (Fig. 3D).

In all TZ, doublet region, and InvC mutants examined, NPHP-2::GFP and ARL-13::GFP still localized to the cilium, suggesting that either unknown factors or redundant pathways are required for establishing ciliary territories. Periciliary mislocalization was observed for both reporters across all mutant backgrounds. This suggests that either a delicate, easily perturbed interaction network is required for NPHP-2 and ARL-13 ciliary import/export, or that the overexpressed reporter constructs are “leaking” out of the cilium in sensitized mutant backgrounds, or a combination of both possibilities.

### NPHP-2 requires its EF-hand for proper localization and function

We next looked to determine which domains of NPHP-2 were required for InvC localization. In mammalian models, several domains in Inversin are required for ciliary targeting and InvC restriction: the ankyrin repeat region, IQ2 domain, and ninein-homologous region (Fig. 4A) [12], [43]. The IQ and ninein homology domains are not conserved in *C. elegans* NPHP-2; only the ankyrin repeat region, hydroxylation motif (S8 Figure), and two nuclear localization signals (NLSs) are conserved (Fig. 4A) [17]. NLS motifs are hypothesized to play a role in ciliary protein import, and are required for ciliary import of the IFT motor KIF17 [9]. NPHP-2 also contains a predicted calcium binding EF-hand which may function in the same calcium detection capacity as the IQ domain of Inversin [17].

We built NPHP-2::GFP constructs missing the EF-hand (NPHP-2-EFΔ::GFP, residues 520–533), NLS1 (NPHP-2-NLS1Δ::GFP, residues 441–446), or NLS2 (NPHP-2-NLS2Δ::GFP, residues 598–603) (Fig. 4A). Full-length NPHP-2::GFP localized to a short proximal region of amphid channel and phasmid cilia as well as IL, CEP, OLQ, amphid channel and phasmid cilia, and the AWC wing cilia (Fig. 4B). Native promoter driven NPHP-2::GFP was not visible in AWB cilia, as reported previously for AWB-specific promoter driven NPHP-2 [13]. NPHP-2-EFΔ::GFP was generally faint or absent in amphid channel, AWC, and phasmid cilia, indicating that the EF-hand is strictly required for normal localization of NPHP-2 in these cell types. NPHP-2-EFΔ::GFP properly localized in IL cilia, indicating that the fluorescent reporter was being synthesized and folded correctly. NPHP-2-EFΔ::GFP was additionally present in either CEP or OLQ cilia, though specific identification was difficult due to their close proximity. Deletion of either NLS1 or NLS2 did not perturb NPHP-2 localization (Fig. 4B).

We next tested whether these domain deletion constructs were functional by attempting to rescue the severe Dyf phenotype of *nphp-2 nphp-4* mutants; *nphp-4* single mutants are nonDyf, allowing for easy determination of rescue [17]. Rescue of the *nphp-2 nphp-4* SynDyf phenotype by NPHP-2-NLS1Δ::GFP and NPHP-2-NLS2Δ::GFP was comparable to rescue by full length NPHP-2::GFP. NPHP-2-EFΔ::GFP only weakly rescued the SynDyf phenotype, indicating that the EF-hand is critical for both normal function and localization of NPHP-2 (Fig. 4C). These results indicate that the calcium-binding EF-hand plays a significant role in both localization and function of NPHP-2 in amphid channel and phasmid cilia, but is dispensable for localization in cilia of CEP, OLQ, and inner labial neurons.

### UNC-119 is associated with the doublet region

**Figure 5:**
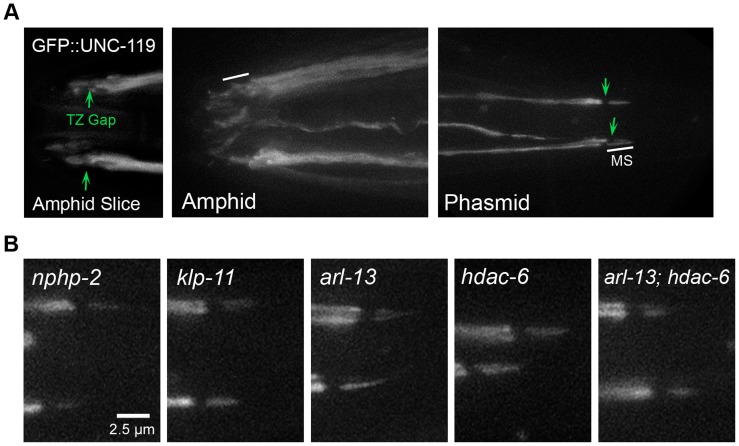
UNC-119 localizes to the proximal cilium in phasmids and does not require DR and InvC genes to target the cilium. (A) N-terminal GFP tagged UNC-119 localizes to dendrites and cilia of amphid channel and phasmid neurons. The TZ gap (arrow) can be discerned in the cilia of amphid channel neurons. In cilia of the CEP and OLQ neurons, GFP::UNC-119 fluorescence was faint and infrequently visible. In phasmid cilia, GFP::UNC-119 localizes to the doublet region, and is excluded from the TZ (arrow) and singlet region. (B) GFP::UNC-119 localization in doublet region- and InvC-associated mutants. GFP::UNC-119 localizes to the proximal cilium in all mutant backgrounds examined.

Mammalian Unc119b localizes to the proximal cilium and is required for the targeting of several proteins to the InvC [26], and *C. elegans unc-119* is required for singlet region biogenesis in amphid cilia [44], suggesting a compartment-specific role. We therefore examined the localization of p*osm-6*::GFP::UNC-119. In phasmid cilia, GFP::UNC-119 localization was similar to NPHP-2::GFP and ARL-13::GFP: its localization was restricted only to a small proximal portion of the cilium and was excluded from the TZ. Similar to localization in phasmid cilia, GFP::UNC-119 likely localized to the doublet region and not the entire cilium of amphid cilia (Fig. 5A). GFP::UNC-119 was not motile in cilia.

We also examined the dependence of UNC-119 localization on doublet region-associated genes. Specific amphid mislocalization was difficult to determine, and we therefore focused on phasmid cilia (S4 Figure). In phasmid cilia, GFP::UNC-119 did not require *nphp-2*, *arl-13*, *hdac-6*, or *klp-11* for ciliary targeting or restriction to the proximal cilium (Fig. 5B).

### IFT motors and *unc-119* genetically interact with doublet region-associated genes

**Figure 6:**
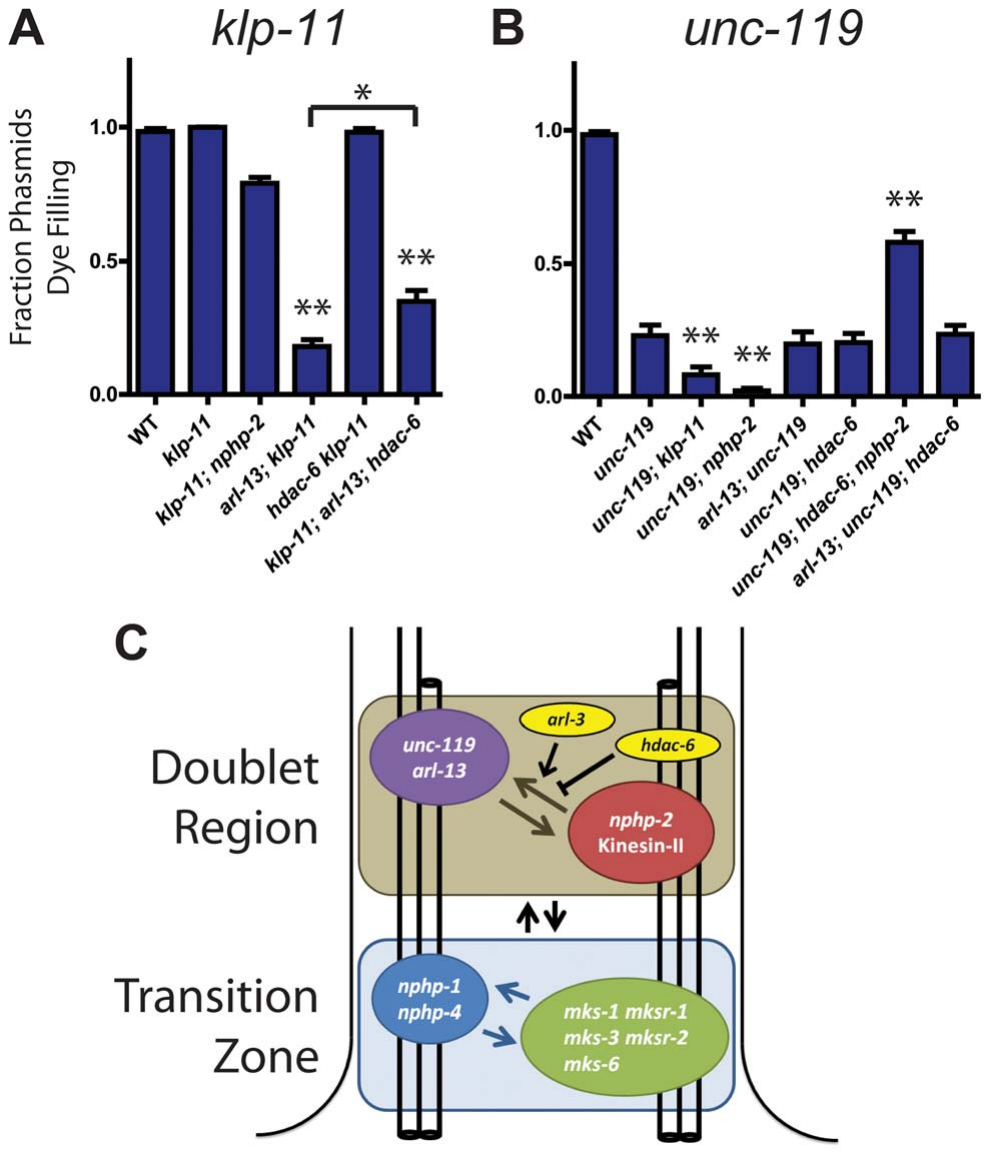
*klp-11* and *unc-119* genetically interact with *arl-13* and *nphp-2* in an *hdac-6* dependent manner. (A) *klp-11* single mutants are not Dyf. *klp-11* is SynDyf with *arl-13*, which is partially suppressed by deletion of *hdac-6*. (B) *unc-119* single mutants are moderately Dyf. *unc-119* is SynDyf with both *klp-11* and *nphp-2*. *unc-119; hdac-6; nphp-2* triple mutants exhibit suppression of the *unc-119* Dyf phenotype. (C) Diagram of interactions between *klp-11*, *arl-13*, *nphp-2*, *unc-119*, and *hdac-6*, and between DR and TZ genes, based on SynDyf phenotypes presented in panels A and B and in Fig. 1 and S5 Figure. The T-bar indicates *hdac-6* mediated suppression of SynDyf phenotypes. Data was analyzed with pairwise Mann-Whitney U-test between wild type, double mutants, and their respective single mutants, followed by the Holm-Bonferroni multiple comparison adjustment. *, significant versus single mutants at a total alpha of 0.05. **, significant versus single mutants at a total alpha of 0.01. *nphp-2*, *arl-13*, *hdac-6*, and *arl-13; hdac-6* Dyf data is presented in Fig. 1.

We examined the genetic interactions between the doublet region-associated genes *nphp-2*, *arl-13*, and their modulators *hdac-6* and *arl-3* with the two IFT motors, *osm-3* and *klp-11*. In both amphids and phasmids, *arl-13; klp-11* was SynDyf [37]. *arl-13; klp-11* dye-filling defects were slightly suppressed by deletion of *hdac-6* (Fig. 6A, S5A Figure). *klp-11; arl-3* double mutants yielded a very mild SynDyf phenotype (S1B Figure). *osm-3* single mutants are missing a singlet region and are completely Dyf, which precludes searching for synthetic interactors of *osm-3*. Instead, we assayed *osm-3* double mutants for suppression of the severe dye-filling defect; in no mutant background examined was the defect suppressed (Fig. 6B, S5B Figure).

We next examined genetic interactions between *unc-119* and other doublet region-associated genes. *unc-119* single mutants were severely Dyf, with the phasmid phenotype being less severe than the amphids (Fig. 6B, S5A Figure) [44]. In phasmids, both *nphp-2* and *klp-11* were SynDyf with *unc-119*. Surprisingly, we found that *hdac-6* suppressed Dyf defects in the *unc-119; nphp-2* double mutant to the level of the *nphp-2* single mutant (Fig. 6B). *arl-3* deletion was also able to significantly suppress the *unc-119* Dyf phenotype in both amphids and phasmids (S1B Figure).

Genetic interactions between *nphp-2*, *arl-13*, *klp-11*, *unc-119*, *hdac-6,* and *arl-3* are summarized in Fig. 6C. Doublet region-associated genes fall into two redundant pathways or modules: *nphp-2*+*klp-11* and *arl-13*+*unc-119*. Deletion of two genes within a module does not result in an increase in Dyf severity over that present in single mutants, but deletion of any two genes from different modules yields a SynDyf phenotype. Interactions between these modules are regulated by *hdac-6* and *arl-3*.

Double mutants with a deletion in a single TZ gene and a single doublet region gene are SynDyf. To determine whether *hdac-6* mediated suppression of SynDyf defects extended to these cross-compartmental genetic interactions, we assayed for suppression of SynDyf defects in *nphp-2 nphp-4* mutants by *hdac-6* and *arl-3*. We found that neither *hdac-6* nor *arl-3* suppressed the severe *nphp-2 nphp-4* SynDyf defect (S5D Figure). This suggests that *hdac-6* functions specifically in doublet region pathways.

### Axonemal glutamylation is downstream of the action of *nphp-2*, *arl-13*, *unc-119*, and *hdac-6*

Post-translational glutamylation predominantly occurs on the C-terminal tails of α- and β-tubulin of axonemal B-tubules [45]–[49], and regulates microtubule stability and IFT motor function [50]. Glutamylation is specifically associated with the doublet region, as B-tubules define and are only present in the doublet region. Additionally, in *Chlamydomonas* and *Paramecium*, TZ microtubules are not glutamylated [45], [51]. In *C. elegans* and vertebrates, mutants with defects in tubulin glutamylation or *arl-13* exhibit B-tubule degeneration [38], [50], [52]. We therefore determined whether doublet region associated genes regulated tubulin glutamylation, or whether tubulin glutamylation specified the localization of doublet region proteins.

**Figure 7:**
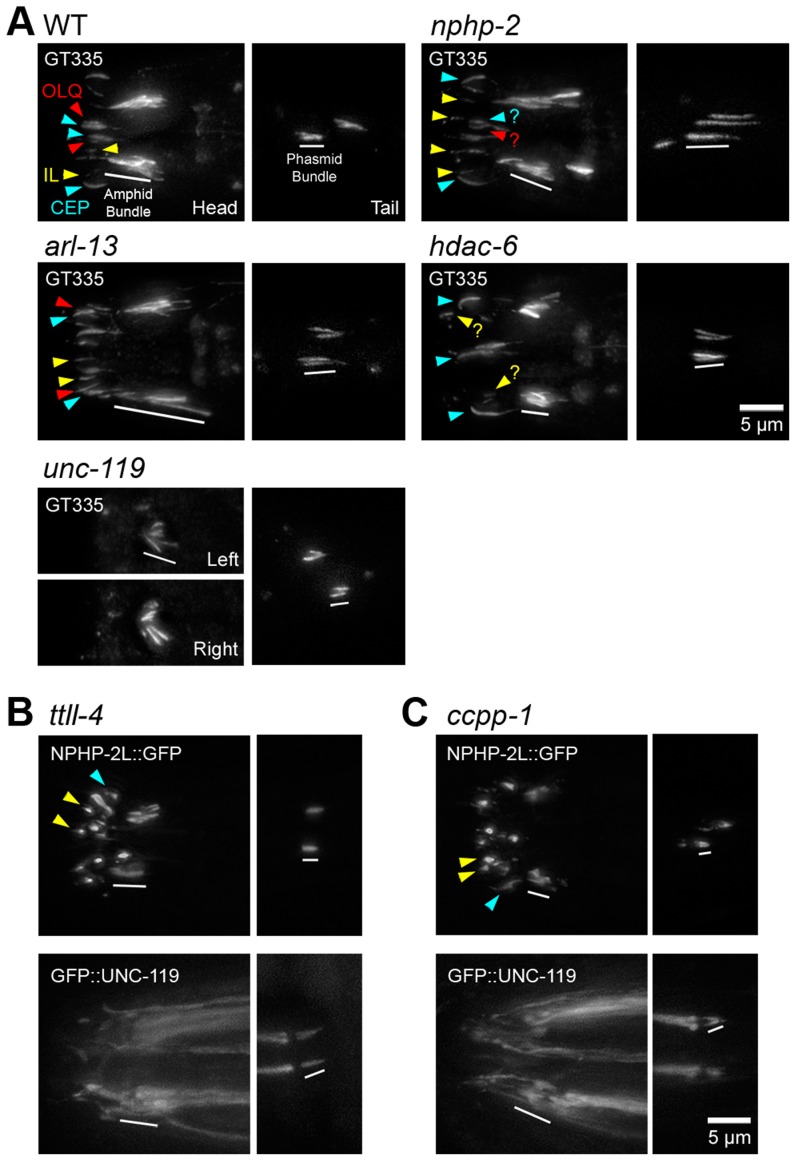
*nphp-2*, *arl-13*, and *hdac-6* regulate glutamylation in head and tail cilia. (A) Worms stained with GT335 anti-glutamylated tubulin antibody. In WT background, GT335 labels amphid and phasmid doublet region microtubules, and CEP doublet region and singlet region microtubules consistently, and OLQ cilia inconsistently. CEP distal microtubule singlets were also labelled by GT335. Inner labial cilia were also glutamylated, but specific IL1 and IL2 identification was not possible. *nphp-2* mutants showed characteristic posterior shifted cilia, and glutamylation in the head looked similar to WT, with infrequent weak inner labial cilia staining. Phasmids exhibited an elongated glutamylation signal. *arl-13* mutants exhibited elongated staining of amphid bundle, CEP, and OLQ cilia. Amphid staining in *unc-119* mutants was extremely shortened and cilia were angled inwards. *unc-119* mutants exhibited almost no staining of CEP, IL, and OLQ cilia. *hdac-6* mutants displayed shorter amphid bundle staining, and reduced IL and OLQ staining. (B) In *ttll-4* mutants, NPHP-2::GFP and GFP::UNC-119 localize similarly to WT. (C) In *ccpp-1* mutants, both NPHP-2::GFP and GFP::UNC-119 localize similarly to in WT, and GFP::UNC-119 is present in the TZ and accumulates at the distal dendrite. Yellow arrowheads – IL2 cilia, red arrowheads - OLQ cilia, blue arrowheads – CEP cilia, white bar – amphid/phasmid bundle.

In wild-type animals, the anti-glutamylated tubulin antibody GT335 labeled the doublet region of amphid channel and phasmid cilia (Fig. 7A) [52]. *nphp-2* mutants exhibited characteristic cilia displacement in the amphids, but no qualitative changes in head cilia glutamylation. The glutamylation signal in *nphp-2* phasmid cilia ranged from wild-type-like to extremely elongated, which is consistent with the TEM observation of B-tubules extending into the distal axoneme (Fig. 2B). *arl-13* mutants exhibited elongated staining in amphid channel cilia. Amphid staining in *unc-119* mutants was extremely shortened and cilia were angled inwards. *hdac-6* mutants appeared to have shortened GT335 staining of amphid channel cilia. We also observed significant differences in the length of the phasmid GT335 ciliary signal. Both *arl-13* (3.95±0.25 µm) and *nphp-2* (4.30±0.32 µm) mutants had phasmid staining significantly longer than in wild type (2.79±0.07 µm), while *unc-119* (2.40±0.05 µm) mutants had staining significantly shorter (S7E Figure). The length of GT335 staining in amphid cilia was not quantified due to the difficulty of unbiased measurement of a single cilium within the amphid bundle.

To determine if doublet region-associated protein localization was dependent on tubulin glutamylation status, we examined the localization of NPHP-2::GFP and GFP::UNC-119 in *ccpp-1* and *ttll-4* mutants. Mutants of *ccpp-1*, which encodes a tubulin deglutamylase, display degenerating amphid channel and phasmid cilia with a concomitant dye-filling defect; this is suppressed by deletion of the opposing glutamylase, encoded by the tubulin tyrosine ligase-like gene *ttll-4* [50]. In each mutant, NPHP-2::GFP and GFP::UNC-119 reporters were targeted to cilia and were restricted to the proximal cilium similarly to wild-type (Fig. 7B,C). This was surprising in the case of *ccpp-1* mutants, as cilia degenerate as the worm ages (Fig. 7B,C). These reporters were still doublet region-associated in earlier larval stages of *ccpp-1* mutants when ciliary degeneration was not as severe (S9C-D Figure). Combined, these results indicate that *nphp-2*, *arl-13*, *unc-119*, and *hdac-6* lie upstream in regulation of tubulin glutamylation pathways, and the localization patterns of their protein products are not defined by tubulin glutamylation.

### Doublet region protein territories are genetically regulated

**Figure 8:**
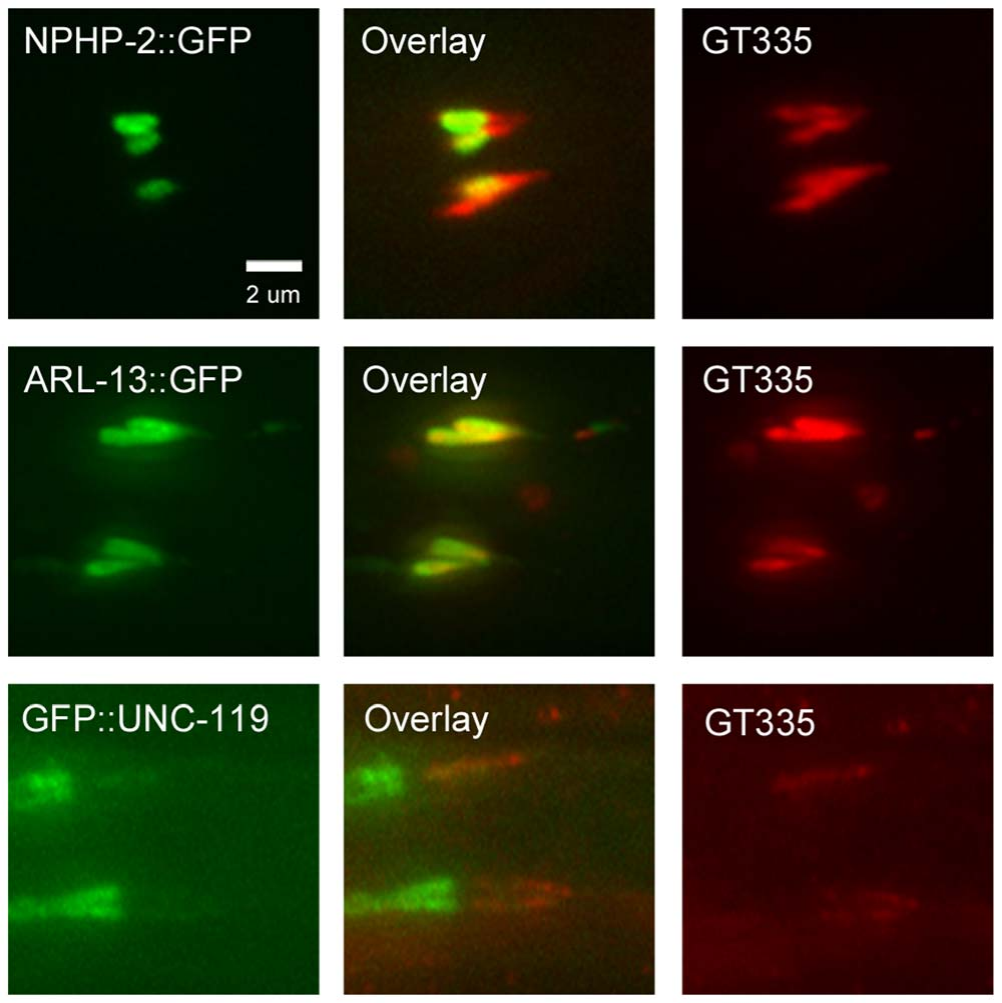
NPHP-2::GFP, ARL-13::GFP, and GFP::UNC-119 colabel with GT335 staining. In amphid channel and phasmid cilia, NPHP-2::GFP, ARL-13::GFP, and GFP::UNC-119 signals overlap with GT335 staining. ARL-13::GFP and GT335 staining overlap completely. NPHP-2::GFP does not overlap completely with GT335. TZ staining by GT335 is frequently visible, as in the NPHP-2::GFP overlay. Though the signal of GFP::UNC-119 is dim due to the staining procedure, GFP::UNC-119 appears not to overlap completely with GT335. Data is quantified in S6A Figure.

To allow for a more direct comparison of subciliary localization, we stained transgenic NPHP-2::GFP, ARL-13::GFP, and GFP::UNC-119 strains with GT335 (Fig. 8). NPHP-2::GFP did not fully overlap with GT335, only colabelling the proximal portion of the doublet region of amphid and phasmid cilia. However, ARL-13::GFP colabelled with a greater portion of the GT335 doublet region signal in amphid cilia than did NPHP-2::GFP, and completely colabelled with GT335 in phasmid cilia (Fig. 8, S6A Figure); this suggests that ARL-13 is associated with the microtubule doublets that define the doublet region. GFP::UNC-119 colabelled with either a significant fraction of the length of the GT335 signal. Additionally, GFP::UNC-119 did not extend beyond the GT335 labelled doublet region, indicating that GFP::UNC-119 is excluded from the TZ, which is not labelled by GT335.

We also examined the territory length of each of the fluorescent reporters as a fraction of the total cilia length. Transgenic animals were incubated with DiI to label the length of the cilium. NPHP-2::GFP marked a significantly shorter fraction of the length of the cilium than did ARL-13::GFP or GFP::UNC-119 (S6C Figure).

To understand how the localization of doublet region-associated proteins is genetically regulated, we measured the length of the cilium marked by NPHP-2::GFP, ARL-13::GFP, GFP::UNC-119, and KAP-1::GFP—a component of Kinesin-II—in different mutant backgrounds (S7 Figure). ARL-13::GFP and NPHP-2::GFP have interdependent localizations: in *arl-13* mutants, the NPHP-2::GFP territory was extended along the cilium, and in *nphp-2* mutants, the ARL-13::GFP territory was extended. *unc-119* mutants exhibited shortened ARL-13::GFP and NPHP-2::GFP territories (S7A-B Figure). Additionally, *klp-11* mutants had a shorter NPHP-2::GFP, but not ARL-13::GFP, localization signal (S7A Figure). No significant differences were found in the territory lengths of GFP::UNC-119 and KAP-1::GFP in any of the mutant backgrounds (S7C-D Figure). The length of the tubulin glutamylation signal is also genetically controlled; the GT335 signal is shortened in *unc-119* mutants, and elongated in *nphp-2* and *arl-13* mutants (S7E Figure).

In sum, the NPHP-2::GFP territory size is shorter than the territories of doublet region components ARL-13 and Kinesin-II, marks a shorter length of the cilium than ARL-13 and UNC-119, is shorter than the doublet region-linked glutamylated tubulin signal, and colabels only a portion of both amphid channel and phasmid GT335 staining. We conclude that NPHP-2 marks a region of the cilium distinct from the doublet region, and propose that this region is analogous to the InvC of mammalian cilia.

## Discussion

In this study, we present evidence that (1) *C. elegans* possesses a conserved InvC compartment, (2) interactions between *nphp-2* and *arl-13* regulate microtubule ultrastructural patterning, (3) InvC and doublet region sizes are distinct and genetically regulated, (4) *hdac-6* and *arl-3* modulate interactions between *nphp-2*, *arl-13*, *klp-11*, and *unc-119*, (5) and that microtubule glutamylation is downstream of the action of InvC and doublet region genes. Additionally, we found that genes associated with the proximal cilium (TZ, InvC, and doublet region) can be grouped into parallel genetic modules, which interact to drive ciliary anchoring and proper ciliogenesis. Finally, we addressed several possible mechanisms for the ciliary targeting and InvC restriction of NPHP-2.

### Limitations of overexpression reporter constructs

In the last twenty years, fluorescent tagging of proteins has allowed for unparalleled insight into *in vivo* localization and transport mechanisms. However, the primary method of introducing transgenes into *C. elegans*, microinjection, yields extrachromosomal arrays containing many copies of the reporter construct [53]. Many aspects of ciliogenesis are tightly regulated, and may require stoichiometric quantities of components for proper function. We previously showed that overexpression of proteins may lead to defects in ciliogenesis and IFT, resulting in the SynDyf phenotype [17], [54].

We sought to minimize these effects on our conclusions by avoiding direct comparisons between multiple reporters, and using antibody colabeling when comparisons were required. In wild-type and mutant backgrounds, we tested all constructs for dominant defects in ciliogenesis (S1 Table). In the respective mutant background, we routinely test for rescue of mutant phenotype, which indicates that the reporter is functional (Fig. 4A) [17], [29], [50], [54]–[57]. For example, NPHP-2::GFP localization is similar in wild-type and *nphp-2* mutant backgrounds, and rescues the *nphp-2 nphp-4* SynDyf phenotype (Fig. 4B) [17]. Therefore we can conclude that NPHP-2::GFP provides and accurate and functional reflection of the endogenous localization pattern.

The use of ARL-13::GFP is well established in the literature, with five papers examining ARL-13::GFP localization in amphid and phasmid cilia [34], [35], [37], [58], [59], and a sixth paper examining ARL-13::GFP localization in AFD cilia [60]. In two papers, the ARL-13::GFP construct is the same as was used in our work [35], [59]. Moreover, this ARL-13::GFP reporter is functional [35] and displays a similar localization in wild-type and *arl-13* backgrounds (Jinghua Hu, personal communication). In three papers, ARL-13::GFP reporters are used to determine subciliary localization of ARL-13::GFP [34], [59], [60]. Additionally, the ARL-13::GFP transgenic lines used in the papers by the Hu and Blacque labs were built independently and exhibit similar localizations.

In the future, genomic engineering (e.g., CRISPR) will allow for easy single-copy fluorescent tagging of proteins and *in vivo* analysis, addressing many of these concerns [61]. These techniques are not a panacea though, as several genes, including *nphp-2*, may be expressed at too low a level for single-copy tagged constructs to be visible without advanced microscopic techniques (personal communication, Knudra Transgenics).

### The nature of the doublet region

**Figure 9:**
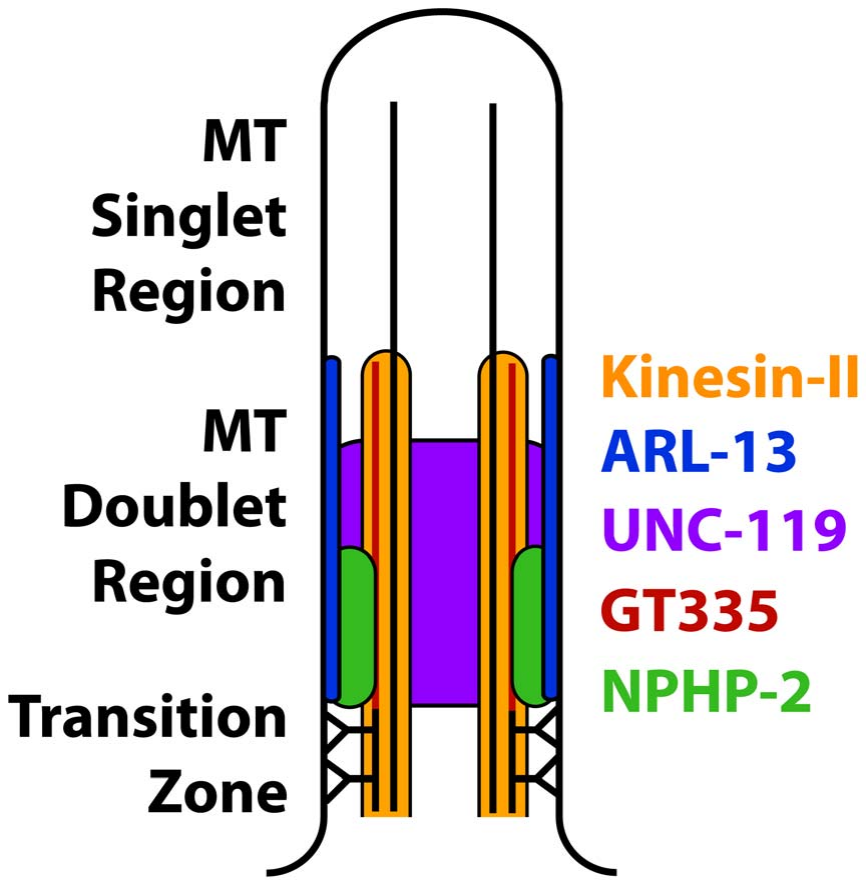
Model of the composition of the proximal cilium. ARL-13 is depicted as membrane associated based on published characterizations [37]. NPHP-2 is depicted as membrane associated based on the membrane association of Inversin in mammalian primary cilia, and because in *C. elegans* NPHP-2 reporters appear membrane associated by casual observation [12], [17]. Kinesin-II is microtubule associated, and UNC-119 is depicted nonspecifically because of the diffuse localization of GFP::UNC-119. Poly-glutamylated tubulin is depicted as a modification of the B-tubule, as reported [45].

The *C. elegans* doublet region and the mammalian proximal InvC have been considered analogous [11], [13], [17], [34], [37]. In *C. elegans*, a number of factors have been associated with the doublet region, including NPHP-2, ARL-13, UNC-119, ARL-3, HDAC-6, the Kinesin-II components KAP-1/KLP-11/KLP-20 [35]–[37], and glutamylated tubulin [50]. However, mammalian orthologs of many *C. elegans* doublet region proteins localize along the entire cilium [26], [62]–[71]; this casts doubt on the equivalence between the *C. elegans* doublet region and the mammalian InvC. It is likely that the *C. elegans* ciliary doublet region is analogous to the entire mammalian ciliary doublet-based cilia shaft, and that the mammalian InvC is analogous to a *C. elegans* InvC (modelled in Fig. 9).

### NPHP-2 localization requires an EF-hand

Calcium signaling plays a crucial role in signal transduction and ciliary function [72], [73]; cilia have high intraciliary calcium concentrations, and many TZ proteins possess calcium binding domains [74]. The NPHP-2 vertebrate homolog, Inversin, has two identified calmodulin-binding IQ domains [75], [76], one of which is required for proper localization [12]. Calmodulin detects intracellular calcium concentrations through a calcium-binding EF-hand. Though *C. elegans* NPHP-2 does not encode a predicted IQ domain, it does possess an EF-hand. This EF-hand is required for the localization and function of NPHP-2 in amphid and phasmid cilia, similarly to the IQ2 domain of Inversin. A significant difference exists between the EF-hand of NPHP-2 and the IQ2 domain of Inversin: deletion of the EF-hand of NPHP-2 results in a complete lack of ciliary localization, whereas deletion of the IQ2 domain of Inversin results in a mislocalization of Inversin along the entire cilium. In both systems, Inversin/NPHP-2 no longer localizes to the InvC. This suggests that Ca^2+^ detection/binding by and the subsequent hypothetical modulation of the activity of Inversin/NPHP-2 is a critical, conserved feature of the protein. Two possibilities arise for the function of these domains: Ca^2+^ specifies the localization of NPHP-2, modulates the activity of the protein, or both.

### UNC-119 is a proximal ciliary protein

We found that UNC-119 localizes to the proximal cilium and is excluded from the distal region. GFP::UNC-119 and GT335 colabel, indicating that GFP::UNC-119 is excluded from the TZ, as TZ microtubules are not glutamylated (See Fig. 8, in which GT335 colabels with TZ-excluded ARL-13::GFP and NPHP-2::GFP). In *C. elegans*, UNC-119 labels a shorter portion of the cilium than ARL-13 or GT335, markers associated with the doublet region. Additionally, mammalian Unc119b physically interacts with the InvC component Nphp3 and is proximally restricted in cilia of RPE cells, suggesting that Unc119b is associated with the InvC [26]. However, GFP::UNC-119 marked a larger portion of the cilium than did NPHP-2::GFP.

The *C. elegans* genome encodes homologs of many of many Unc119b shuttle proteins, including Unc119b, Arl3, and RP2, and two myristoylated ciliary proteins which require *unc-119* for ciliary localization [77]. *arl-3* genetically interacts with *unc-119* and *nphp-2*, suggesting that in *C. elegans* the components of the shuttle are in place. These shuttle components do not appear to be required for the localization of NPHP-2, as NPHP-2 is imported into the cilium in *unc-119* and *arl-3* mutants. In *unc-119* mutants, NPHP-2 exhibited a unique distal dendritic localization pattern that cannot be attributable to TZ leakage; it is unknown whether this population represents NPHP-2 that has not been properly imported into the cilium or is mistargeted NPHP-2.

### The function of *hdac-6*

In both mammalian systems and *C. elegans*, Hdac6/*hdac-6* functions as an antagonist of ciliogenesis and cilia stability [16], [62]. In mammalian primary cilia, Hdac6 functions as an α-tubulin K40 deacetylase regulates microtubule function and stability [62], [78]–[81]. The *C. elegans* genome encodes a single α-tubulin with the acetylatable residue K40, MEC-12. However, there is no direct evidence for *mec-12* expression in amphid and phasmid neurons, and the anti-α-tubulin-K40 antibody 6-11b-1 does not label amphid and phasmid cilia in wild-type animals or *hdac-6* mutants (S10 Figure) [82]. Alternative tubulin acetylation sites may exist, including on β-tubulin [83]; *hdac-6* could deacetylate these secondary sites. NPHP-2 contains a predicted N-terminal acetylation site (at 2S) which may be deacetylated by HDAC-6; this may modulate binding between NPHP-2 and its targets [84]. Additionally, HDAC-6 may have other unidentified ciliary targets [35]. Determining the mechanism by which HDAC-6 acts as a genetic modifier of InvC and doublet region gene defects is an important future direction.

### Doublet region and InvC components modulate tubulin post-translational modification

Tubulin glutamylation is associated with the proximal portions of microtubule B-tubules in *C. elegans* cilia, mouse spermatozoa flagella, and *Chlamydomonas* flagella [47], [50], [85]. In *C. elegans*, microtubule glutamylation is linked to microtubule ultrastructure, stability, and maintenance [50], [86]. Multiple doublet region genes regulate microtubule glutamylation, and we observed a correlation between ectopic glutamylation and ectopic microtubule doublets. Additionally, ciliary targeting of doublet region proteins is not dependent on glutamylation status. Therefore, *nphp-2*, *arl-13*, *hdac-6*, and *unc-119* function upstream of microtubule glutamylation, which may enable them to exert an influence on microtubule patterning, IFT, ciliogenesis, and, in the case of *unc-119*, singlet region biogenesis. These pathways may be conserved: in Arl13b/*hennin* mutant mice, ciliary microtubule glutamylation intensity is reduced, and microtubule B-tubules have ultrastructural defects [38], [64].

### Doublet region- and InvC-associated genes form genetic modules

In *C. elegans*, TZ-associated genes can be grouped into two distinct, partially redundant genetic and physical modules [8], [17], [42]. Doublet region- and InvC-associated genes may be grouped in a similar manner into a *nphp-2*+*klp-11* module and an *arl-13*+*unc-119* module. *hdac-6* appears to function outside the two modules, negatively regulating both: deletion of *hdac-6* in SynDyf double mutants suppresses the SynDyf phenotype. In mammalian primary cilia, Hdac6 also plays an antagonistic role, destabilizing cilia through deacetylation of tubulin, a pathway suppressed by Inversin [16]. *arl-3* may function outside of the two modules in a cell-type specific manner; in phasmids it genetically interacts with components from both modules, but in amphids *arl-3* only genetically interacts with only the *arl-13*+*unc-119* module and not the *nphp-2*+*klp-11* module. In amphid cilia, *nphp-2* also does not interact with the TZ SynDyf network.

Curiously, a further two genetic module organization exists between TZ genes and the InvC/doublet region genes. *nphp-2 nphp-4, mks-3; nphp-2,* and *arl-13; nphp-4* double mutants exhibit severe ciliogenic defects [17]. Deletion of one TZ gene from either TZ module and one doublet region gene from either doublet region module yields a SynDyf phenotype, though not all combinations have been tested. The TZ SynDyf network and the doublet region SynDyf network can be thought of as two genetically interacting “super-modules”, each consisting of two to three sub-modules described in this and previous work [8], [17], [25], [42].

### Origin of the Inversin Compartment

The localization requirements for multiple InvC localizing components have been previously determined, but how the InvC is initially established is not known.

In *C. elegans*, the InvC is likely established early in cilia development, as NPHP-2 is proximally restricted in phasmid cilia as early as the first larval L1 stage immediately following hatching (S9A Figure), and is not motile (S9B Figure), unlike the larval stage-dependent dynamic localization of ARL-13 [34]. We have eliminated several mechanisms for the establishment of the InvC. Ciliary ultrastructure does not seem to play a role, as in both mammals and *C. elegans*, the localization of Inversin/NPHP-2 is associated with only a sub-portion of the doublet region where there are no identifiable ultrastructural features [12]. Tubulin post-translational modifications also do not appear to specify the InvC, as *nphp-2* (and genetically interacting doublet region components) lies upstream of glutamylation pathways. The TZ does not appear to play a major role in specifying the InvC. NPHP-2 still localizes and is restricted to the proximal cilium in TZ single and double mutants. IFT is another candidate mechanism, but we found that although IFT components genetically interact with InvC and doublet region associated genes, Kinesin-II is not required for NPHP-2 localization. In mammalian cilia, the Unc119b shuttle is required for the ciliary import of Nphp3; this activity is upstream of the action of Inversin in Nphp3 localization. In *C. elegans* phasmid cilia, NPHP-2 does not require either *unc-119* or its effector *arl-3* for ciliary import or InvC restriction (S2A Figure).

Several mechanisms for establishing the InvC remain. First, calcium may play a role. Both Inversin and NPHP-2 require a calcium binding domain for InvC localization [12]; the origin and nature of the calcium signal these domains detect is unknown. Second, the InvC may also be initially established by a diffusion of factors from the cilia base. A third possibility is that cilia membrane composition may help define the InvC. The cilium has a distinct membrane composition from the plasma membrane, and the different ciliary subregions may also have differential membrane composition.

### Final summary

We propose that the logic underlying the establishment of the NPHP-2/Inversin compartment may be similar in *C. elegans* and mammals, in a manner independent of microtubule ultrastructure. We have shown that doublet region- and InvC-associated genes interact to guide ciliogenesis, cilia placement, cilia ultrastructure, protein composition, and tubulin post-translational modification. The next challenge is to determine what initially patterns the doublet region and InvC, and to understand the function of these cilia regions.

## Materials and Methods

### General molecular biology

Standard protocols were followed for all molecular biological procedures. PCR amplification using Taq polymerase (New England BioLabs, Ipswich, MA, USA) was used for genotyping deletion alleles, and was followed by restriction digest for SNP diagnosis. PCR amplification for construction of transgenic constructs was performed with Phusion High fidelity DNA polymerase (New England BioLabs), templated off *C. elegans* genomic DNA. Sequencing was performed offsite (GeneWiz, South Plainfield, NJ, USA). PCR primer and construct sequences are available upon request.

### Bioinformatics and computer tools

Protein BLAST was used to find sequence orthologs [87]. All protein sequence information other than that of *C. elegans* was provided by NCBI, and all *C. elegans* nucleotide and protein sequences were provided by WormBase (Releases WS229 and WS234). Structural motif and domain predictions were generated by MotifScan [88]. Acetylation motifs were identified using NetAcet 1.0 [84]. Coiled-coil regions were identified with COILS [89]. ApE 2.0.36 was used for sequence manipulation, annotation, and restriction site identification.

### Strains and maintenance

All strains were cultured at room temperature, unless otherwise noted, under standard conditions [90]. Transgenic strains using *pha-1* selection were grown at 25°C, and *pha-1(e2123)* mutants were grown at 15°C. Deletion alleles were outcrossed to *him-5* at least four times. Strains used in this study are listed in S2A Table. Alleles used were as follows: *nphp-2(gk653), nphp-4(tm925), mks-3(tm2547), arl-13(gk513), unc-119(ed3), hdac-6(ok3203), arl-3(tm1703), him-5(e1490), osm-3(p802), klp-11(tm324), ccpp-1(ok1821), and ttll-4(tm3310).* Primers used for diagnosis are listed in S2B Table. All transgenic strains used in Fig. 1–9 were tested using dye-filling for dominant negative defects in ciliogenesis. We observed no adverse effects in NPHP-2::GFP and GFP::UNC-119 transgenic strains but did find dominant negative defects in ARL-13::GFP transgenic strains (S1 Table).

The full length isoform of NPHP-2 was used in all NPHP-2 reporter constructs. The full length isoform, NPHP-2L, differs from the shorter isoform, NPHP-2S, in that the shorter isoform is missing 22 non-conserved amino acids of unknown function. Both isoforms have similar subciliary localization [17].

### Electron microscopy

*nphp-2* and *arl-13; hdac-6; nphp-2* young adult animals were fixed using 3.5% glutaraldehyde +1% PFA in 0.1M HEPES and then in 1% OsO4+1.25% K4Fe(CN)4 in 0.1M HEPES. Samples were infiltrated and embedded in Embed-812 plastic resin. *arl-13; nphp-2* young adult animals were fixed using high-pressure freeze fixation and freeze substitution in 2% OsO4+2% water in acetone as the primary fixative [91]. Samples were slowly freeze substituted in an RMC freeze substitution device, before infiltration with Embed-812 plastic resin. Images for wild-type animals fixed by a comparable immersion fixation method (cf. [32]) are now curated by the Hall lab at Einstein courtesy of E. Hedgecock. These wild type images are also available online at www.wormimage.org.

For TEM, serial sections (70 nm thickness) of fixed animals were collected on copper slot grids coated with formvar and evaporated carbon and stained with 4% uranyl acetate in 70% methanol, followed by washing and incubating with aqueous lead citrate. Images were captured on a Philips CM10 transmission electron microscope at 80 kV with a Morada 11 megapixel TEM CCD camera driven by iTEM software (Olympus Soft Imaging Solutions).

For each strain, we imaged three individuals that were fixed chemically. Additionally, we were concerned that the severe defects seen in the *arl-13; nphp-2* double mutant were partially due to the harsh chemical fixation method. We fixed a fourth double mutant using high pressure freeze (HPF) fixation, which introduced fewer artifacts to confirm the validity of the chemical fixation data. We used the same strain in the construction of the double *arl-13; nphp-2* and triple *arl-13; hdac-6; nphp-2* mutants as was used in the previously published EM of the *arl-13* single mutant.

### Imaging

Animals were mounted on 5% Noble agar pads on standard microscope slides, and immobilized with a five minute incubation in 10 mM sodium azide. Worms were imaged using a Zeiss Plan-AXIOCHROMA 100X 1.4NA oil objective on a Zeiss Axio Imager.D1M (Zeiss, Oberkochen, Germany) with a Retiga-SRV Fast 1394 digital camera (Q-Imaging, Surrey, BC, Canada). Exposure time for antibodies was 100 ms, and exposure time for GFP fluorophores was 250 ms. Images were captured and manipulated using Metamorph software (Version 7.6.1.0, MDS Analytical technologies, Sunnyvale, CA, USA). Image stacks were 3D deconvolved using Auto Deblur software (Version 1.4.1, Media Cybernetics, Bethesda, MD, USA). Figures and diagrams were created with Adobe Photoshop CS3 (Version 10.0, Adobe Systems, San Jose, CA, USA) and Adobe Illustrator (Version 13.0.0, Adobe Systems). Image brightness and contrast were modified uniformly across an image, but gamma was not adjusted from 1.00. Brightness manipulations are similar, but not identical, across panels and figures. Significant variations in absolute intensity are noted where appropriate. For all strains, unless noted, worms were picked at L4 stage 24 hours before imaging.

### Statistical analysis

All statistical analysis was performed with a combination of GraphPad Prism (Version 5.01, GraphPad Software, La Jolla, CA, USA) and Microsoft Excel (Version 14.0.7106, 32-Bit, Microsoft Corporation, Seattle, WA, USA). Sample size (*n*) for all figures is listed in S3 Table. Minimum p value for significance was set at 0.01 for all analyses unless otherwise specified. All parametric and continuous data types were analyzed using unpaired t-tests with Welch's correction to avoid assumption of equal variance. When multiple t-tests were performed on related data sets presented together, the Holm-Bonferroni multiple comparison adjustment was used to ensure the total alpha for the analysis did not exceed 0.01. All nonparametric and discontinuous data types were analyzed using Mann-Whitney U-test. Similar to the analysis of continuous data types, the Holm-Bonferroni multiple comparison adjustment was employed to ensure total alpha for all related comparisons did not exceed 0.01. Specific pairwise comparisons made are described in figure legends. Letters on graphs indicate statistically distinct groups, e.g., all groups marked ‘A’ are significantly different from all groups marked ‘B’.

### Dye-filling assays

Staged young adult hermaphrodites were washed of plates with M9, and then rinsed three further times in M9, using gentle centrifugation to pellet the worms between rinses. Worms were then incubated in 40 µg/mL DiI (2.5 mg/mL dimethyl formamide stock, diluted 1∶1000 in M9) (Invitrogen) for 30 minutes in the dark. Worms were then rinsed three times in M9 as before, and were then placed on a seeded plate for a further 30-60 minutes to recover and flush dye from the digestive tract. Animals were anesthetized with 10 mM sodium azide and then immediately scored on a compound microscope (see Imaging section) using for dye-filling by manual counting of filled cell bodies. Cell body counts within the amphid or phasmid organs were averaged together to yield the average number of cells filling per organ per worm, and subjected to statistical analysis. Ectopically dye-filled neurons (e.g., IL2s) were not included in the total count.

### Antibody staining

Antibody staining was performed under the standard Ruvkun-Finney protocol (Anatomical Methods, [92]) using GT335, an antibody against branch point single and polyglutamylated tubulin. Both primary (GT335, mouse, 1∶100, Enzo Life Sciences) and secondary antibody (Alexa Flour 568 goat anti-mouse, 1∶2000 dilution, Life Technologies) washes were performed at 4°C overnight. Young adult hermaphrodites were selected for imaging using the number of eggs in the animal—between one and ten—as a proxy for age.

## Supplemental Information

S1 Figure *arl-3* genetically interacts with InvC and doublet region associated genes. Dye filling was used to test for synthetic interactions between InvC and doublet region genes. (A) *arl-3* interacts with *nphp-2* and *arl-13* in a sensillum-specific manner. *arl-3* single mutants and *arl-3; hdac-6* double mutants are nonDyf in amphids and phasmids. In both amphids and phasmids, *arl-13; arl-3* double mutants are mildly SynDyf, which is suppressed by *hdac-6* deletion. *arl-3* deletion did not suppress *arl-13; nphp-2* defects. *hdac-6* mediated suppression of *arl-13; nphp-2* defects requires *arl-3*. In phasmids, but not amphids, *nphp-2* genetically interacted with *arl-3*, but this was not suppressed by *hdac-6* deletion. (B) *arl-3* genetically interacts with *unc-119* and *klp-11* in amphids and phasmids. *arl-3* deletion moderately suppressed *unc-119* single mutant Dyf. *klp-11; arl-3* was slightly SynDyf. Data in panel A was statistically analyzed in conjunction with data from Fig. 1 because of the overlap in genotypes examined. Both panel A and Fig. 1 were analyzed with pairwise Mann-Whitney U-test between all groups, followed by the Holm-Bonferroni multiple comparison adjustment with a total alpha of 0.01. Genotypes from either panel A of this figure or Fig. 1 sharing a capital letter are not significantly different, whereas groups from either panel with different capital letters do differ significantly. Data panel B was analyzed using pairwise Mann-Whitney U-test between double mutants, their respective single mutants, and wild type followed by the Holm-Bonferroni multiple comparison adjustment with a total alpha of 0.01. **, double mutant phenotype is significantly different from both respective single mutants.
(TIF)

S2 Figure Localization requirements of NPHP-2::GFP and ARL-13::GFP in phasmid cilia. (A) NPHP-2::GFP does not require *mks-3; nphp-4*, *hdac-6*, or *arl-13; hdac-6* for ciliary targeting or restriction to the proximal cilium. (B) Contrast enhanced version of Fig. 3C-D. ARL-13::GFP mislocalizes to the periciliary compartment in TZ and doublet region mutants.
(TIF)

S3 Figure NPHP-2 and ARL-13 do not require TZ-, doublet region-, and InvC-associated genes for ciliary targeting in amphids. (A) Localization of NPHP-2::GFP is subtly disrupted in *mks-3*, *nphp-4* and *mks-3; nphp-4* mutants. Periciliary puncta in amphid channel cilia and altered staining in IL2, CEP, and OLQ cilia are visible. (B) The NPHP-2::GFP amphid bundle is shortened in *klp-11* and *unc-119* mutants, and elongated in *arl-13* mutants. Periciliary puncta are visible in *klp-11* and *arl-13* mutants. *hdac-6* deletion does not suppress the *arl-13* phenotype. (C) ARL-13::GFP localizes primarily to the doublet region of amphid channel cilia, and to a nonspecific proximal region of IL2 cilia. In *mks-3* and *nphp-4* mutants, ARL-13::GFP localization in amphid channel cilia looks grossly wild-type. CEP and OLQ cilia show increased ARL-13::GFP localization. (D) *unc-119* mutants exhibit distal dendritic accumulation of ARL-13::GFP. *klp-11* and *nphp-2* mutants exhibit increased CEP and OLQ ARL-13::GFP localization. Arrowheads indicate periciliary puncta. Bar indicates distal dendritic localization.
(TIF)

S4 Figure Amphid UNC-119 localization in InvC and doublet region mutants. GFP::UNC-119 appears similar in wild-type (Fig. 5A), *nphp-2,* and *hdac-6* amphid channel cilia. *klp-11* and *arl-13* exhibit an accumulation of GFP::UNC-119 in amphid channel, OLQ, CEP, and inner labial cilia. *hdac-6* deletion does not suppress GFP::UNC-119 mislocalization in *arl-13* mutants. Green arrow – TZ gap, yellow arrowheads – IL2 cilia, red arrowheads - OLQ cilia, blue arrowheads – CEP cilia, white bar – amphid bundle.
(TIF)

S5 Figure Amphid dye-filling of IFT and *unc-119* mutants. (A) *klp-11* single mutants are not Dyf. *klp-11* is SynDyf with *arl-13*, which is suppressed by deletion of *hdac-6*. (B) *unc-119* single mutants are severely Dyf. *hdac-6; nphp-2* suppresses the *unc-119* Dyf phenotype to a small degree. (C) *osm-3* single mutants are both severely amphid and phasmid Dyf. In no double mutant was this suppressed. (D) *hdac-6; nphp-2 nphp-4* and *arl-3; nphp-2 nphp-4* worms were assayed for suppression of SynDyf defects. Neither strain exhibited significant suppression as compared to the *nphp-2 nphp-4* double mutant. Data was analyzed with pairwise Mann-Whitney U-test between wild type, double mutants, triple mutants and their respective single mutants, followed by the Holm-Bonferroni multiple comparison adjustment. **, significant versus single mutants at a total alpha of 0.01.
(TIF)

S6 Figure NPHP-2::GFP marks a significantly smaller region of the cilium than ARL-13::GFP and GFP::UNC-119. (A) Worms expressing NPHP-2::GFP, ARL-13::GFP, and GFP::UNC-119 reporters were stained with GT335. The ratio of the length of each reporter localization pattern to the length of the entire cilium, minus the TZ was computed per-cilium. NPHP-2::GFP marks a significantly shorter proportion of the cilium than either ARL-13::GFP or GFP::UNC-119. (B) Absolute lengths of data presented in panel A. (C) Worms expressing NPHP-2::GFP, ARL-13::GFP, and GFP::UNC-119 reporters were incubated with DiI to label cilia. The ratio of the length of each reporter localization pattern to the length of the entire cilium, minus the TZ was computed per-cilium. NPHP-2::GFP marks a significantly shorter proportion of the cilium than either ARL-13::GFP or GFP::UNC-119. (D) Absolute lengths of data presented in panel C. Phasmid cilia lengths in transgenic strains as measured using DiI staining, and reporter localization size. Data in each panel was analyzed with pairwise t-tests with Welch's Correction, followed by the Holm-Bonferroni multiple comparison adjustment for a total alpha of 0.01.
(TIF)

S7 Figure Doublet region- and InvC-associated genes regulate the localization patterns of doublet region and InvC components. Each data point represents the averaged lengths of the GFP signal or immunofluorescence in all visible and distinct phasmid cilia within a single animal. (A) NPHP-2::GFP localization length is significantly decreased in *klp-11* and *unc-119* mutants, and significantly increased in *arl-13* mutants. *hdac-6* deletion partially suppresses the *arl-13* phenotype. (B) ARL-13::GFP localization length is increased in *nphp-2* and decreased in *unc-119* mutants. (C) GFP::UNC-119 localization length does not change significantly in any strain. (D) KAP-1 localization length measurements were more variable that the other reporters due to a faint KAP-1::GFP signal, but were not significantly different from wild type in any strain. Localization length was significantly altered in *nphp-2* mutants. (E) *nphp-2* and *arl-13* had significantly longer GT335 signals than in wild type. *nphp-2* mutants have increased variability in GT335 signal lengths compared to WT. (F) Diagram representing genetic control of doublet region protein localization in phasmid cilia. An arrow between a gene and a protein/GT335 indicates that the gene is required for the protein/GT335 to label its entire territory. A T-bar between a gene and a protein/GT335 indicates that the gene restricts the size of the protein territory/GT335 signal length. Data was analyzed with an unpaired t-test with Welch's Correction against wild type, followed by the Holm-Bonferroni multiple comparison adjustment for all comparisons in a given panel. **p<0.01.
(TIF)

S8 Figure NPHP-2 contains a strongly conserved hydroxylation motif and a predicted acetylation site. (A) Hydroxylation motif is highlighted in green. Hydroxylated asparagine is highlighted in blue. ANKS6 and INVS alignment are from [24]. (B) NPHP-2 domain model. NPHP-2 contains a predicted acetylation site at 2S, and two predicted SUMOylation sites. Green letters indicate modified residues. Acetylation and SUMOylation motifs do not appear to be conserved.
(TIF)

S9 Figure (A) NPHP-2::GFP localizes to the proximal portion of the cilium in L1 animals. (B) Kymograph of NPHP-2::GFP in phasmid cilia. No movement of NPHP-2::GFP was detectable. Total duration of recording was 40 seconds. (C) NPHP-2::GFP localizes to amphid and phasmid cilia in L1 and L4 stage *ccpp-1* mutants. (D) GFP::UNC-119 localizes to amphid and phasmid cilia in L1 and L4 stage *ccpp-1* mutants. White bars indicate ciliary GFP::UNC-119 localization. White arrow indicates abnormal cilia base accumulation of GFP::UNC-119.
(TIF)

S10 Figure The anti-acetylated tubulin antibody 6-11b-1 does not label amphid channel and phasmid cilia in either WT animals or *hdac-6* mutants. 6-11b-1 antibody labels only dendrites of the touch neurons. *hdac-6* deletion does not increase the amount of acetylation detected in touch neurons. Non-specific labelling is seen in the buccal cavity, and not in amphid cilia, as determined by analysis of 3D image stacks.
(TIF)

S1 Table Transgenic strains used in this study have mild to no ciliogenic defects. Worms from each transgenic strain imaged or otherwise quantified in Fig. 1-9—other than strains with a Dyf or SynDyf background—were assayed for dye-filling defects to determine if there were any gross defects in ciliogenesis or cilia placement. Scores reported are average fraction of cell bodies taking up dye, plus/minus std. err. Yellow cells mark possible dominant negative defects. Transgenic strains expressing NPHP-2::GFP and GFP::UNC-119 did not exhibit defects, but strains expressing ARL-13::GFP all exhibited mild defects.
(XLSX)

S2 Table List of strains and PCR deletion diagnosis primers used in this work. Strains are organized by the figures in which they appear.
(XLS)

S3 Table Experimental sample sizes for figures with statistical analysis.
(XLSX)
