# Supplementary figures and images for: The nphp-2 and arl-13 Genetic Modules Interact to Regulate Ciliogenesis and Ciliary Microtubule Patterning in C. elegans
Source: PLoS Genet. 2014 Dec 11;10(12):e1004866. doi: 10.1371/journal.pgen.1004866 (PMC4263411; doi:10.1371/journal.pgen.1004866)

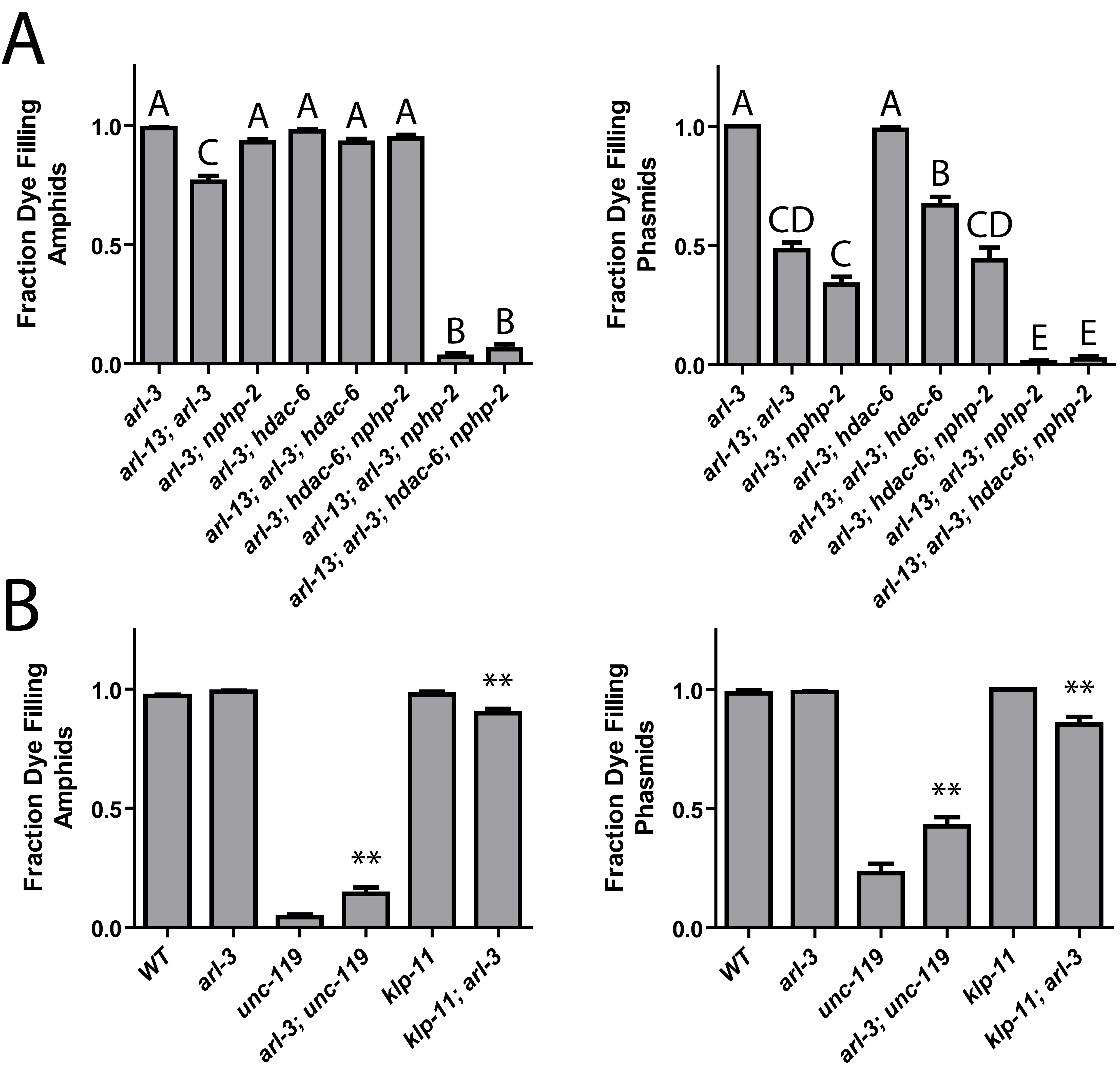

Supplement: S1 Figure — arl-3 genetically interacts with InvC and doublet region associated genes. Dye filling was used to test for synthetic interactions between InvC and doublet region genes. (A) arl-3 interacts with nphp-2 and arl-13 in a sensillum-specific manner. arl-3 single mutants and arl-3; hdac-6 double mutants are nonDyf in amphids and phasmids. In both amphids and phasmids, arl-13; arl-3 double mutants are mildly SynDyf, which is suppressed by hdac-6 deletion. arl-3 deletion did not suppress arl-13; nphp-2 defects. hdac-6 mediated suppression of arl-13; nphp-2 defects requires arl-3. In phasmids, but not amphids, nphp-2 genetically interacted with arl-3, but this was not suppressed by hdac-6 deletion. (B) arl-3 genetically interacts with unc-119 and klp-11 in amphids and phasmids. arl-3 deletion moderately suppressed unc-119 single mutant Dyf. klp-11; arl-3 was slightly SynDyf. Data in panel A was statistically analyzed in conjunction with data from Fig. 1 because of the overlap in genotypes examined. Both panel A and Fig. 1 were analyzed with pairwise Mann-Whitney U-test between all groups, followed by the Holm-Bonferroni multiple comparison adjustment with a total alpha of 0.01. Genotypes from either panel A of this figure or Fig. 1 sharing a capital letter are not significantly different, whereas groups from either panel with different capital letters do differ significantly. Data panel B was analyzed using pairwise Mann-Whitney U-test between double mutants, their respective single mutants, and wild type followed by the Holm-Bonferroni multiple comparison adjustment with a total alpha of 0.01. **, double mutant phenotype is significantly different from both respective single mutants. (TIF) [file pgen.1004866.s001.tif]

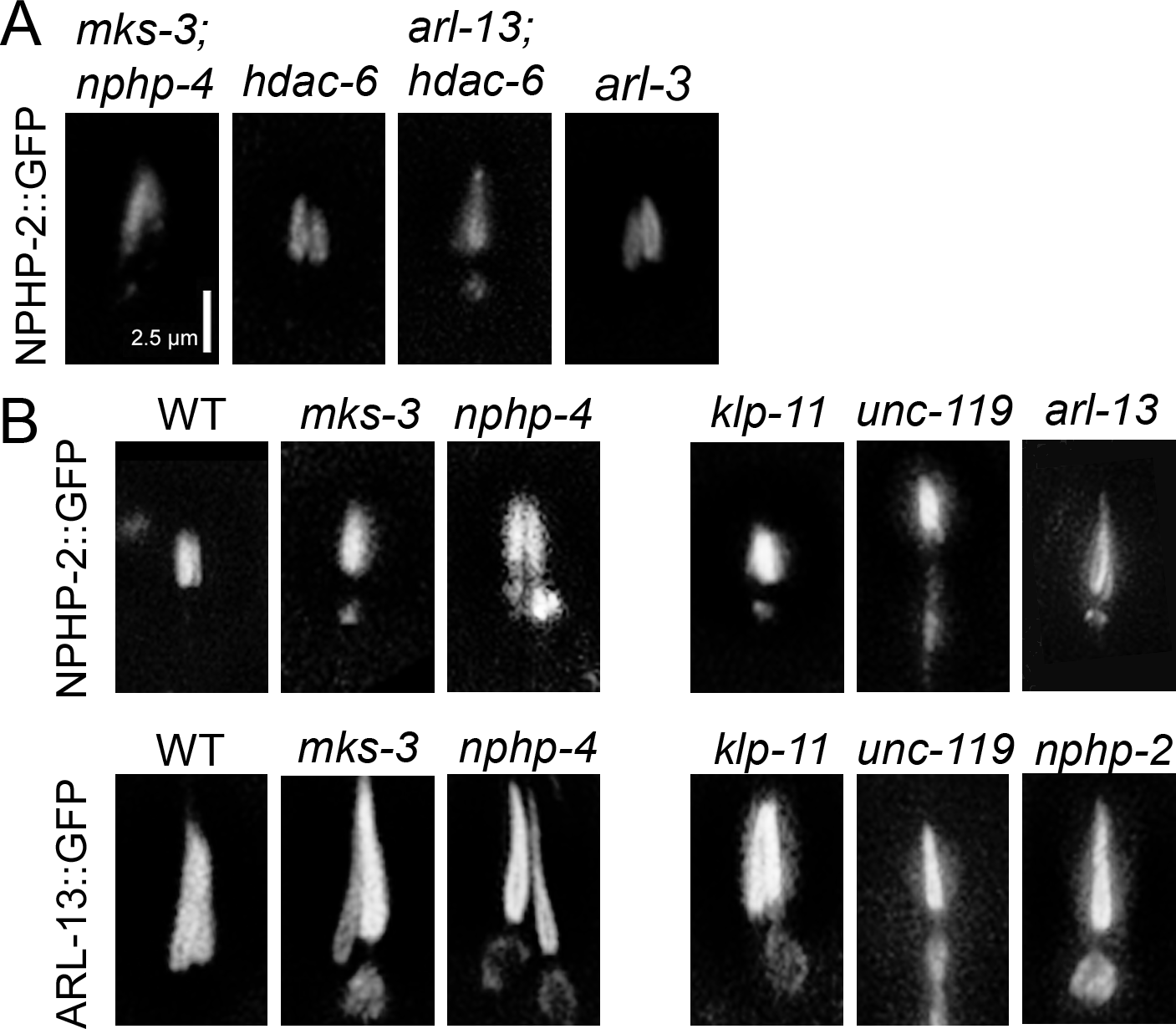

Supplement: S2 Figure — Localization requirements of NPHP-2::GFP and ARL-13::GFP in phasmid cilia. (A) NPHP-2::GFP does not require mks-3; nphp-4, hdac-6, or arl-13; hdac-6 for ciliary targeting or restriction to the proximal cilium. (B) Contrast enhanced version of Fig. 3C-D. ARL-13::GFP mislocalizes to the periciliary compartment in TZ and doublet region mutants. (TIF) [file pgen.1004866.s002.tif]

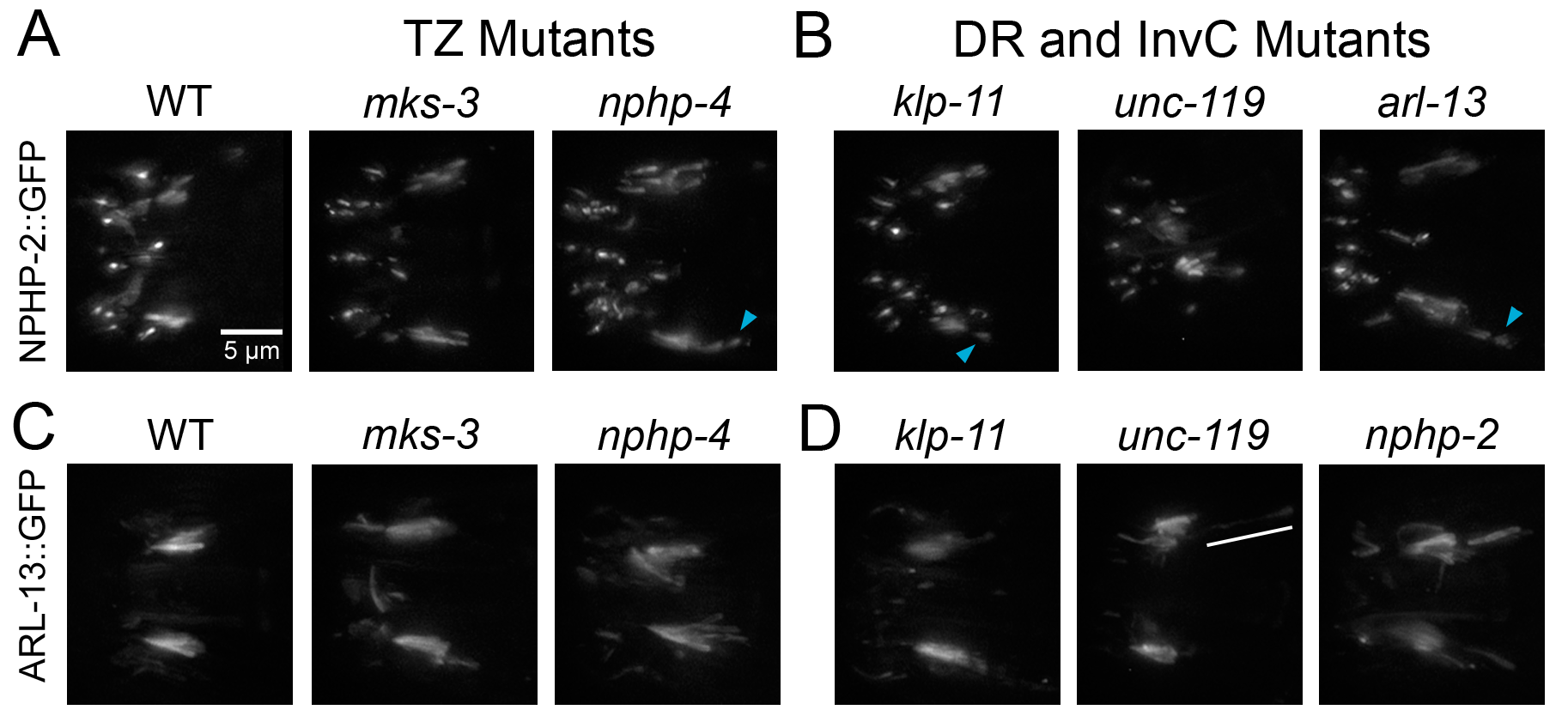

Supplement: S3 Figure — NPHP-2 and ARL-13 do not require TZ-, doublet region-, and InvC-associated genes for ciliary targeting in amphids. (A) Localization of NPHP-2::GFP is subtly disrupted in mks-3, nphp-4 and mks-3; nphp-4 mutants. Periciliary puncta in amphid channel cilia and altered staining in IL2, CEP, and OLQ cilia are visible. (B) The NPHP-2::GFP amphid bundle is shortened in klp-11 and unc-119 mutants, and elongated in arl-13 mutants. Periciliary puncta are visible in klp-11 and arl-13 mutants. hdac-6 deletion does not suppress the arl-13 phenotype. (C) ARL-13::GFP localizes primarily to the doublet region of amphid channel cilia, and to a nonspecific proximal region of IL2 cilia. In mks-3 and nphp-4 mutants, ARL-13::GFP localization in amphid channel cilia looks grossly wild-type. CEP and OLQ cilia show increased ARL-13::GFP localization. (D) unc-119 mutants exhibit distal dendritic accumulation of ARL-13::GFP. klp-11 and nphp-2 mutants exhibit increased CEP and OLQ ARL-13::GFP localization. Arrowheads indicate periciliary puncta. Bar indicates distal dendritic localization. (TIF) [file pgen.1004866.s003.tif]

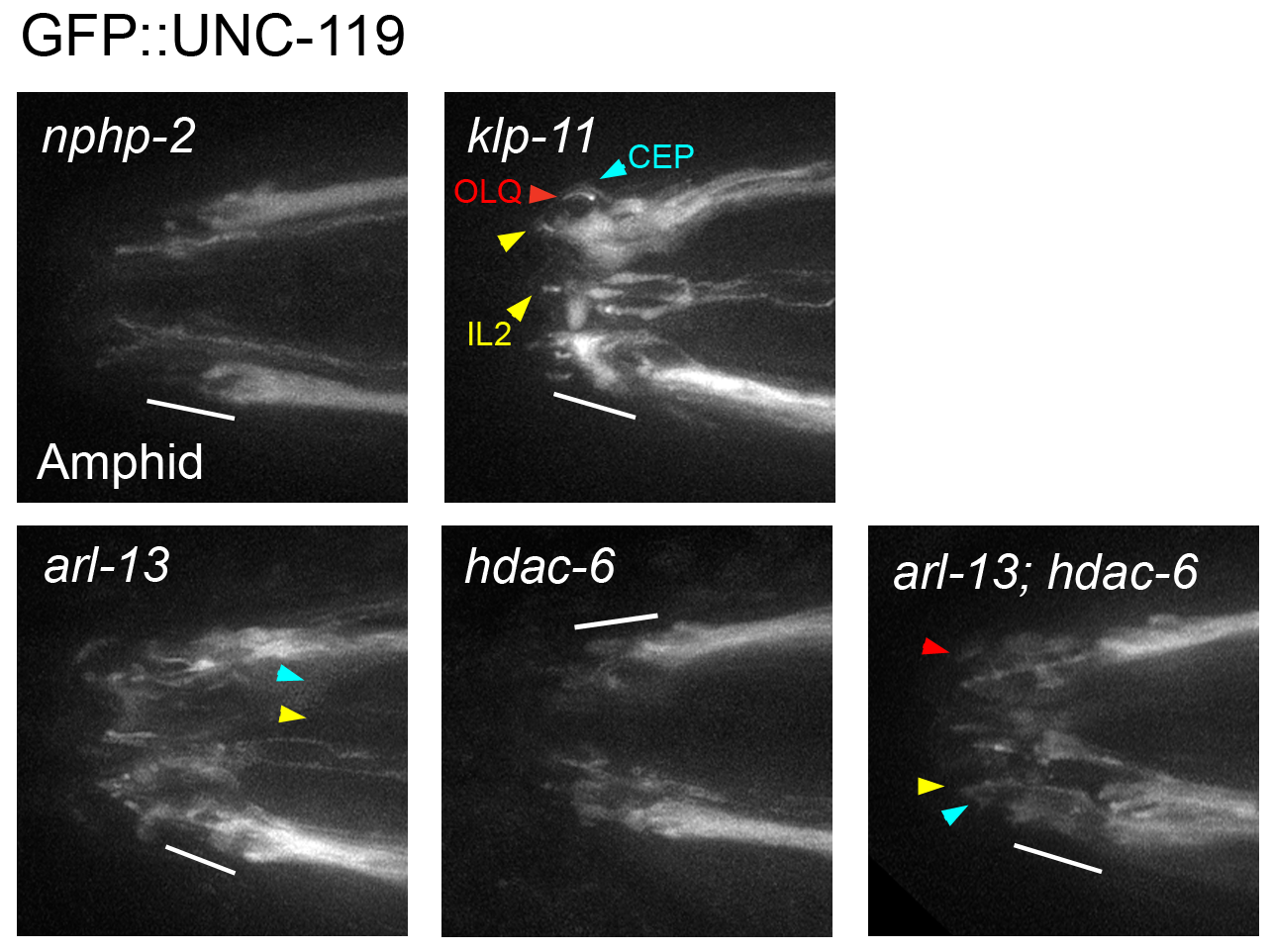

Supplement: S4 Figure — Amphid UNC-119 localization in InvC and doublet region mutants. GFP::UNC-119 appears similar in wild-type (Fig. 5A), nphp-2, and hdac-6 amphid channel cilia. klp-11 and arl-13 exhibit an accumulation of GFP::UNC-119 in amphid channel, OLQ, CEP, and inner labial cilia. hdac-6 deletion does not suppress GFP::UNC-119 mislocalization in arl-13 mutants. Green arrow – TZ gap, yellow arrowheads – IL2 cilia, red arrowheads - OLQ cilia, blue arrowheads – CEP cilia, white bar – amphid bundle. (TIF) [file pgen.1004866.s004.tif]

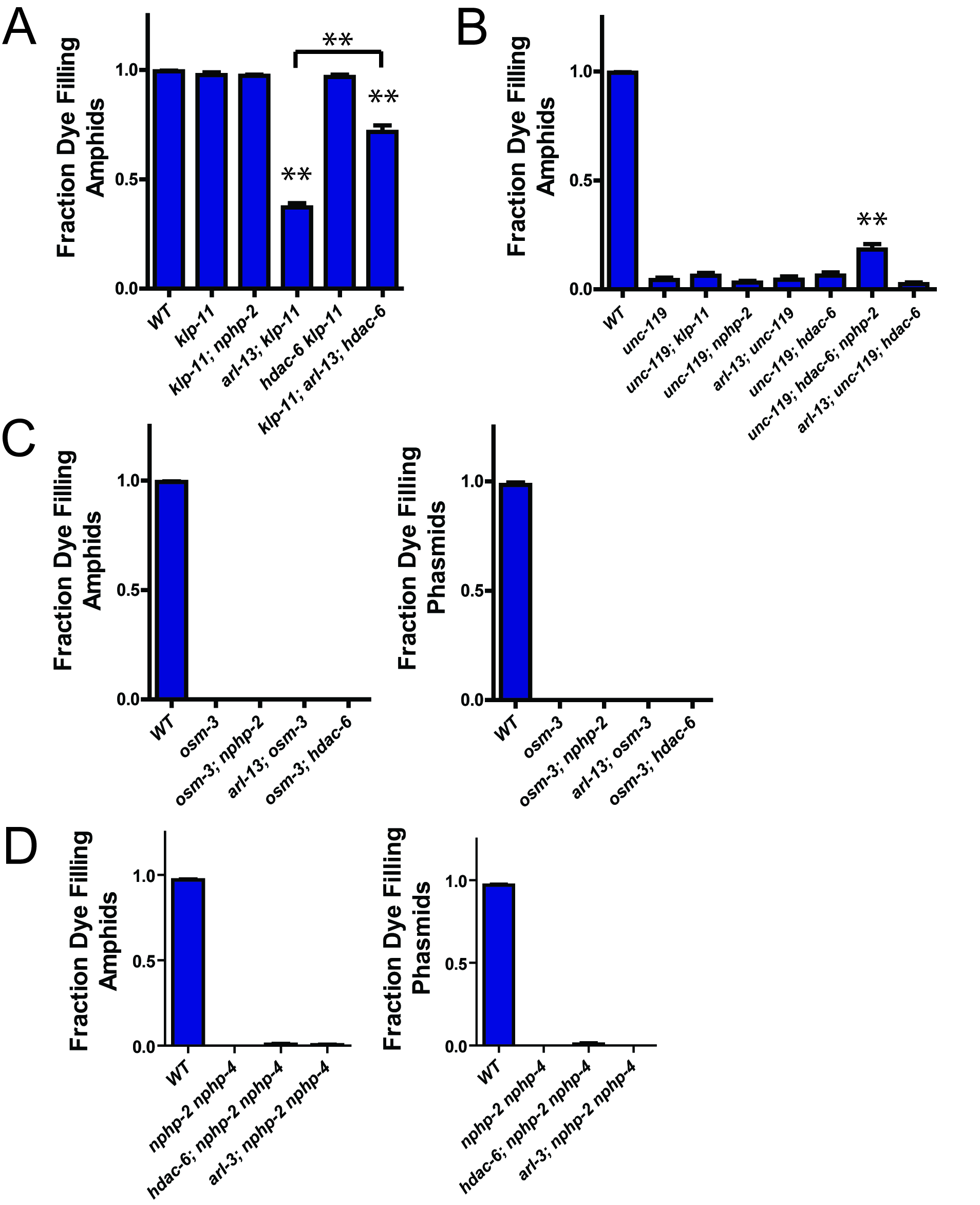

Supplement: S5 Figure — Amphid dye-filling of IFT and unc-119 mutants. (A) klp-11 single mutants are not Dyf. klp-11 is SynDyf with arl-13, which is suppressed by deletion of hdac-6. (B) unc-119 single mutants are severely Dyf. hdac-6; nphp-2 suppresses the unc-119 Dyf phenotype to a small degree. (C) osm-3 single mutants are both severely amphid and phasmid Dyf. In no double mutant was this suppressed. (D) hdac-6; nphp-2 nphp-4 and arl-3; nphp-2 nphp-4 worms were assayed for suppression of SynDyf defects. Neither strain exhibited significant suppression as compared to the nphp-2 nphp-4 double mutant. Data was analyzed with pairwise Mann-Whitney U-test between wild type, double mutants, triple mutants and their respective single mutants, followed by the Holm-Bonferroni multiple comparison adjustment. **, significant versus single mutants at a total alpha of 0.01. (TIF) [file pgen.1004866.s005.tif]

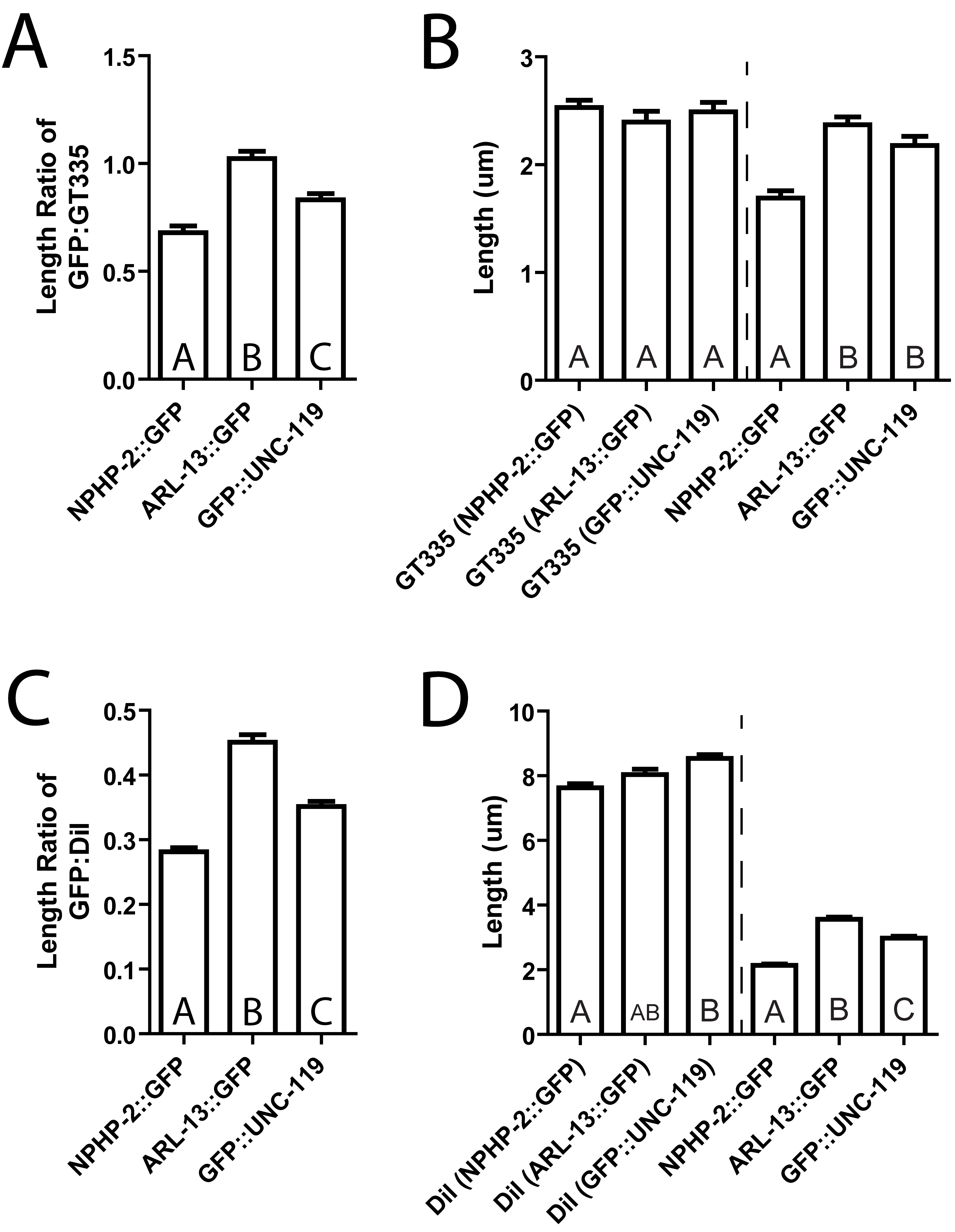

Supplement: S6 Figure — NPHP-2::GFP marks a significantly smaller region of the cilium than ARL-13::GFP and GFP::UNC-119. (A) Worms expressing NPHP-2::GFP, ARL-13::GFP, and GFP::UNC-119 reporters were stained with GT335. The ratio of the length of each reporter localization pattern to the length of the entire cilium, minus the TZ was computed per-cilium. NPHP-2::GFP marks a significantly shorter proportion of the cilium than either ARL-13::GFP or GFP::UNC-119. (B) Absolute lengths of data presented in panel A. (C) Worms expressing NPHP-2::GFP, ARL-13::GFP, and GFP::UNC-119 reporters were incubated with DiI to label cilia. The ratio of the length of each reporter localization pattern to the length of the entire cilium, minus the TZ was computed per-cilium. NPHP-2::GFP marks a significantly shorter proportion of the cilium than either ARL-13::GFP or GFP::UNC-119. (D) Absolute lengths of data presented in panel C. Phasmid cilia lengths in transgenic strains as measured using DiI staining, and reporter localization size. Data in each panel was analyzed with pairwise t-tests with Welch's Correction, followed by the Holm-Bonferroni multiple comparison adjustment for a total alpha of 0.01. (TIF) [file pgen.1004866.s006.tif]

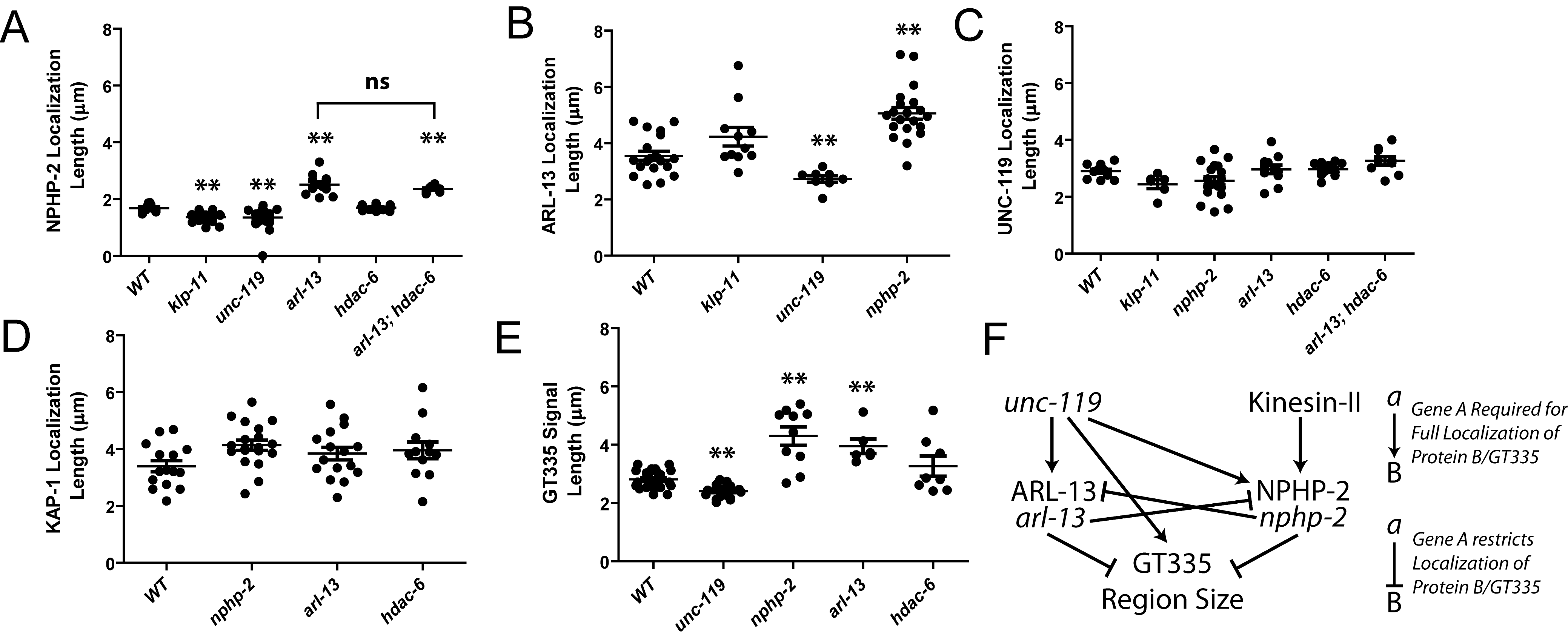

Supplement: S7 Figure — Doublet region- and InvC-associated genes regulate the localization patterns of doublet region and InvC components. Each data point represents the averaged lengths of the GFP signal or immunofluorescence in all visible and distinct phasmid cilia within a single animal. (A) NPHP-2::GFP localization length is significantly decreased in klp-11 and unc-119 mutants, and significantly increased in arl-13 mutants. hdac-6 deletion partially suppresses the arl-13 phenotype. (B) ARL-13::GFP localization length is increased in nphp-2 and decreased in unc-119 mutants. (C) GFP::UNC-119 localization length does not change significantly in any strain. (D) KAP-1 localization length measurements were more variable that the other reporters due to a faint KAP-1::GFP signal, but were not significantly different from wild type in any strain. Localization length was significantly altered in nphp-2 mutants. (E) nphp-2 and arl-13 had significantly longer GT335 signals than in wild type. nphp-2 mutants have increased variability in GT335 signal lengths compared to WT. (F) Diagram representing genetic control of doublet region protein localization in phasmid cilia. An arrow between a gene and a protein/GT335 indicates that the gene is required for the protein/GT335 to label its entire territory. A T-bar between a gene and a protein/GT335 indicates that the gene restricts the size of the protein territory/GT335 signal length. Data was analyzed with an unpaired t-test with Welch's Correction against wild type, followed by the Holm-Bonferroni multiple comparison adjustment for all comparisons in a given panel. **p<0.01. (TIF) [file pgen.1004866.s007.tif]

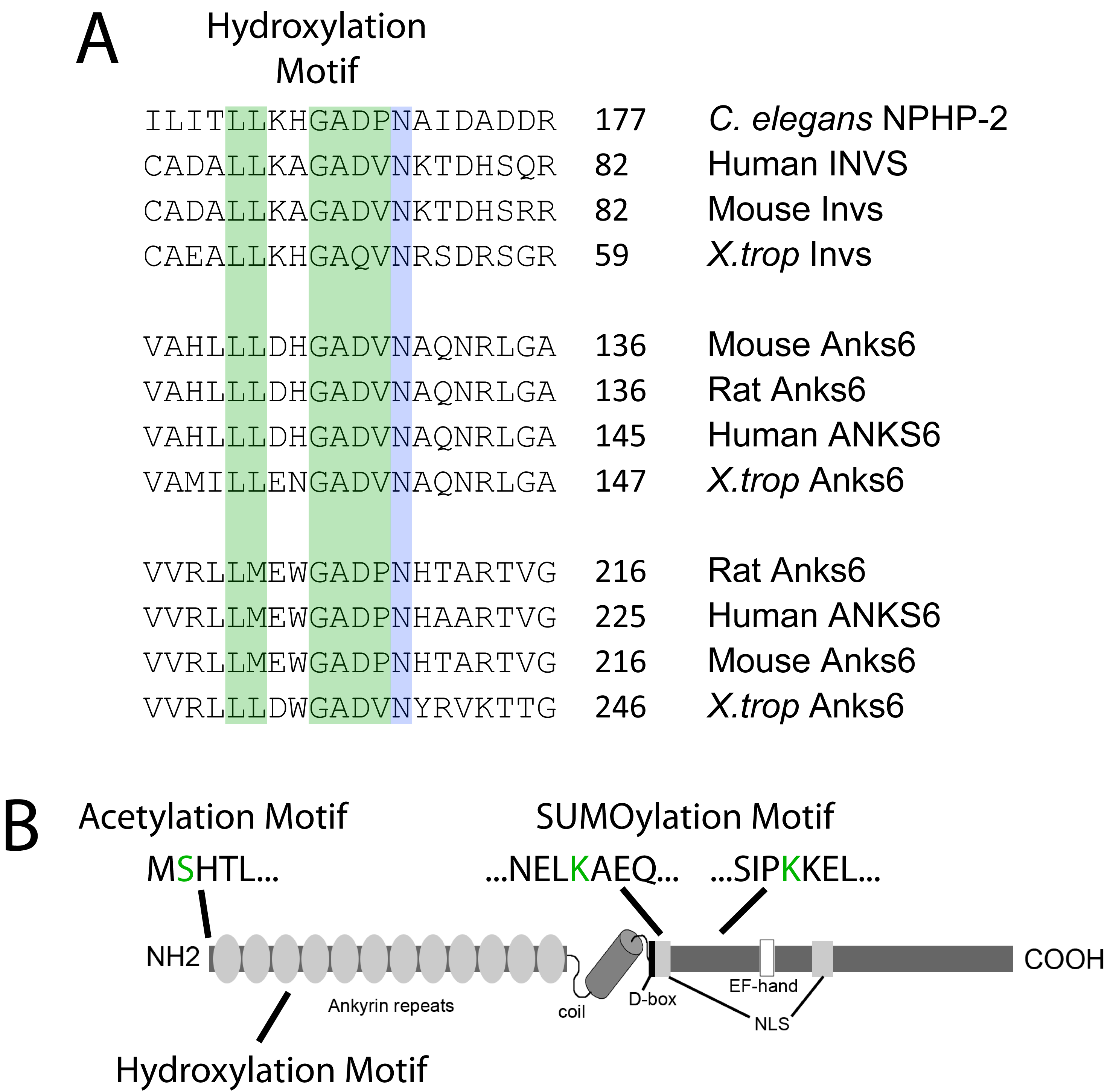

Supplement: S8 Figure — NPHP-2 contains a strongly conserved hydroxylation motif and a predicted acetylation site. (A) Hydroxylation motif is highlighted in green. Hydroxylated asparagine is highlighted in blue. ANKS6 and INVS alignment are from [24]. (B) NPHP-2 domain model. NPHP-2 contains a predicted acetylation site at 2S, and two predicted SUMOylation sites. Green letters indicate modified residues. Acetylation and SUMOylation motifs do not appear to be conserved. (TIF) [file pgen.1004866.s008.tif]

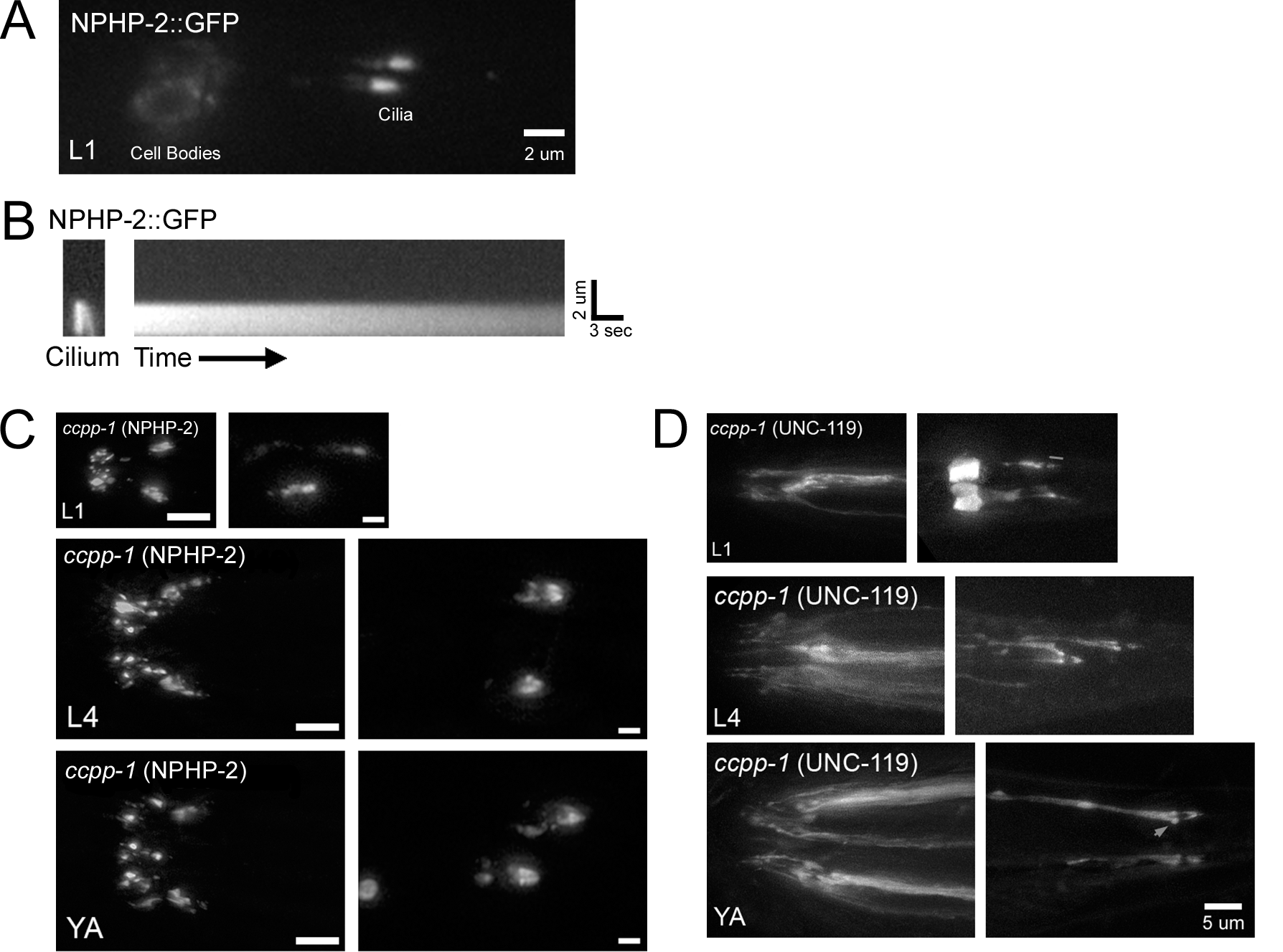

Supplement: S9 Figure — (A) NPHP-2::GFP localizes to the proximal portion of the cilium in L1 animals. (B) Kymograph of NPHP-2::GFP in phasmid cilia. No movement of NPHP-2::GFP was detectable. Total duration of recording was 40 seconds. (C) NPHP-2::GFP localizes to amphid and phasmid cilia in L1 and L4 stage ccpp-1 mutants. (D) GFP::UNC-119 localizes to amphid and phasmid cilia in L1 and L4 stage ccpp-1 mutants. White bars indicate ciliary GFP::UNC-119 localization. White arrow indicates abnormal cilia base accumulation of GFP::UNC-119. (TIF) [file pgen.1004866.s009.tif]

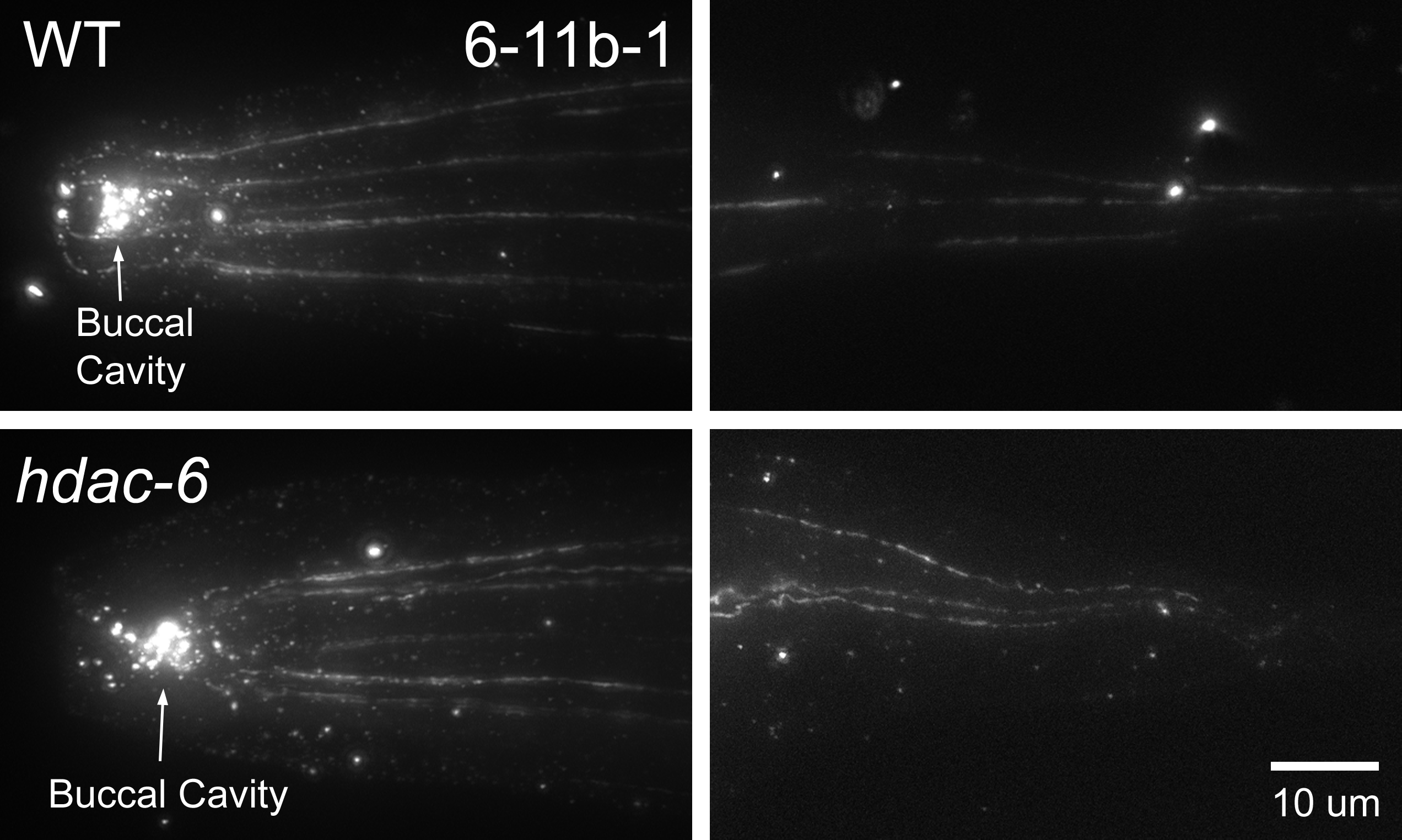

Supplement: S10 Figure — The anti-acetylated tubulin antibody 6-11b-1 does not label amphid channel and phasmid cilia in either WT animals or hdac-6 mutants. 6-11b-1 antibody labels only dendrites of the touch neurons. hdac-6 deletion does not increase the amount of acetylation detected in touch neurons. Non-specific labelling is seen in the buccal cavity, and not in amphid cilia, as determined by analysis of 3D image stacks. (TIF) [file pgen.1004866.s010.tif]
